# Co-manifestation of swallowing dysfunction indicators and vocal fold motion impairment within a shared neural substrate in patients with brain injury: a cross-sectional study

**DOI:** 10.3389/fneur.2026.1790412

**Published:** 2026-06-24

**Authors:** Shujuan Huang, Lirong Liu, Yiming Chen, Chunlong Liu, Si Chen, Hanji Chen, Churong Liu, Hanbo Chen

**Affiliations:** 1 Department of Rehabilitation Therapy, Guangdong Sanjiu Brain Hospital, Guangzhou, China; 2 Department of Rehabilitation, Guangdong Work Injury Rehabilitation Hospital, Guangzhou, China; 3 Clinical Medical College of Acupuncture Moxibustion and Rehabilitation, Guangzhou University of Chinese Medicine, Guangzhou, China

**Keywords:** brain injury, cross-sectional study, dysphagia, fiberoptic endoscopic evaluation of swallowing, vocal fold motion impairment

## Abstract

**Background:**

Vocal fold motion impairment (VFMI) is a common but frequently overlooked complication in patients with brain injury and can significantly compromise multiple airway protective functions—including glottic closure during swallowing, cough reflex, and cough effectiveness—leading to increased risks of aspiration, pneumonia, and poor clinical outcomes. Although swallowing dysfunction and VFMI often coexist in patients with brain injury, the specific characteristics and underlying mechanisms of their association have not been systematically investigated.

**Methods:**

This single-center cross-sectional study consecutively enrolled 280 patients with brain injury from June 2024 to May 2025. All patients underwent fiberoptic endoscopic evaluation of swallowing (FEES) to simultaneously assess 18 swallowing indicators covering 5 dimensions and vocal fold motor function. VFMI was defined as complete fixation or hypomotility of the vocal folds under endoscopy. Multivariable logistic regression analysis identified swallowing indicators that significantly co-occurred with VFMI after adjusting for potential confounders. To address the extreme collinearity between bilateral pyriform sinus secretion variables (Spearman *r* = 0.974), a composite maximum-score variable [PS_MAX = max(left, right)] was constructed.

**Results:**

Among 280 patients, 220 (78.6%) had VFMI. After adjusting for confounders, restricted pharyngeal wall movement (OR = 2.16, 95% CI: 1.06–4.38, *p* = 0.034) and pyriform sinus secretion pooling (PS_MAX: OR = 2.165, 95% CI: 1.067–4.396, *p* = 0.032) consistently co-manifested with VFMI, suggesting their co-occurrence as expressions of shared pharyngolaryngeal neural substrate impairment.

**Conclusion:**

Restricted pharyngeal wall movement and pyriform sinus secretion pooling co-manifest with VFMI as integrated expressions of a shared neural injury substrate—specifically, the overlapping brainstem circuits including the nucleus ambiguus and related pathways that co-regulate pharyngeal constrictor function and vocal cord motor control—rather than as independently co-occurring phenomena. VFMI should be regarded as part of the broader swallowing and airway-protective dysfunction spectrum—encompassing both degglutitive impairment and compromised airway-protective functions including cough reflex and cough effectiveness—caused by brainstem network impairment, rather than an isolated laryngeal disorder. These findings have direct clinical implications in settings where vocal fold status is not simultaneously available, supporting more integrated evaluation and rehabilitation planning that addresses swallowing, cough, and airway-protective function.

## 1 Introduction

Brain injury is one of the leading causes of death and disability worldwide, with a particularly prominent disease burden in the Chinese population (1). Brain injury is frequently accompanied by multi-system dysfunction, among which VFMI, as an often overlooked yet clinically consequential complication, can significantly compromise key airway protective functions—including glottic closure during swallowing, cough reflex, and cough effectiveness—thereby increasing the risk of aspiration pneumonia, malnutrition, and mortality (2). Beyond voice impairment, VFMI is commonly associated with compromised swallowing safety, exerting profound effects on patients’ quality of life and long-term prognosis (3). Therefore, systematic understanding of the functional characteristics of VFMI and its associated factors in patients with brain injury is of great significance for refining clinical assessment and management.

Swallowing function refers to the complex neuromuscular coordination process of safely and effectively transporting food or liquid from the oral cavity to the stomach, involving multiple phases including the oral, pharyngeal, and esophageal phases (4). This process relies on the precise coordination between the brainstem swallowing center and cranial nerve nuclei (particularly the nucleus ambiguus and nucleus tractus solitarius), and is highly sensitive to the temporal activation of pharyngolaryngeal muscles and sensory-motor feedback (5). Dysphagia exhibits a high incidence in neurological diseases such as brain injury and is significantly associated with aspiration, malnutrition, prolonged hospitalization, and increased mortality (6). Multiple swallowing function indicators obtained through fiberoptic endoscopic evaluation of swallowing (FEES), such as secretion management capacity, pharyngeal wall movement, protective reflexes, and residue severity, can intuitively reflect airway protection and pharyngeal clearance capacity, providing important information for clinical understanding of the pathophysiological basis of dysphagia (7).

From anatomical and neurophysiological perspectives, swallowing and vocal cord movement are regulated by common brainstem neural pathways and frequently exhibit comorbidity characteristics in patients with brain injury (8). Previous studies suggest that dysphagia and VFMI commonly coexist in patients with stroke and other neurological diseases, with some studies observing a markedly elevated incidence of dysphagia in patients with vocal cord dysfunction, suggesting that the two may share similar neural injury substrates (9). However, considerable heterogeneity exists across studies regarding the strength of association and specific manifestations: for instance, in some patients with unilateral or low-level lesions, restricted vocal cord movement does not always completely correlate with aspiration or dysphagia severity, suggesting that VFMI may be a critical node within a multifactorial dysfunction network rather than a sole determinant (10–12).

In recent years, related research has gradually shifted from “whether an association exists” toward more refined descriptions of functional characteristics, yet existing evidence still has several limitations (13). First, most studies focus on only a single swallowing dimension (such as aspiration grading or secretion management), lacking systematic integration of multidimensional swallowing function characteristics (14–16); second, substantial variation exists across studies in assessment tools and outcome definitions, increasing uncertainty in result interpretation (17, 18); additionally, some studies employ aspiration or pneumonia as surrogate endpoints without directly assessing vocal cord movement status itself, potentially underestimating the direct association between swallowing dysfunction and VFMI (13, 19, 20).

Based on the above background, this study employs a cross-sectional design with consecutive enrollment at a single specialized hospital to simultaneously assess multidimensional swallowing function indicators and vocal cord movement status through the same fiberoptic endoscopy in patients with brain injury, systematically exploring the association patterns between the two. By integrating multiple standardized indicators including secretion management, swallowing physiology mechanisms, protective reflexes, residue severity, and aspiration risk within a unified FEES assessment framework, this study aims to identify key swallowing function characteristics closely associated with VFMI, thereby deepening understanding of the comorbidity characteristics of post-brain injury dysphagia and VFMI, and providing evidence for conducting more comprehensive functional assessments in clinical practice, with particular attention to identifying the concrete clinical scenarios in which these co-manifestation patterns carry actionable value for detecting VFMI in settings where vocal fold status is not simultaneously available.

## 2 Materials and methods

### 2.1 Study design and study population

This study employed a cross-sectional design and consecutively enrolled 280 patients with brain injury who presented to Guangdong Sanjiu Brain Hospital from June 2024 to May 2025. Inclusion criteria comprised: confirmed diagnosis of brain injury (including intracerebral hemorrhage, cerebral infarction, traumatic brain injury, or brain tumor, diagnosed based on head CT or MRI imaging results); stable vital signs; completion of fiberoptic endoscopic evaluation of swallowing; and signed informed consent from the patient or legal guardian. Exclusion criteria included: prior history of laryngeal surgery (such as vocal cord surgery, laryngeal tumor resection, thyroid surgery, etc.); primary laryngeal diseases (such as laryngeal cancer, laryngeal papilloma, chronic laryngitis); history of organic esophageal lesions (such as post-esophageal cancer surgery, esophageal stricture); and patients with incomplete clinical data.

During the study period, 316 patients with brain injury were screened using consecutive enrollment, whereby all patients meeting preliminary inclusion criteria during the study period underwent systematic assessment. After excluding 36 patients with incomplete clinical data, 280 patients were ultimately included in the analysis. All assessments were completed on the same day during the patients’ stable condition period—specifically, within 48–72 h of admission to our rehabilitation center (not 48–72 h from initial disease onset, as these patients were predominantly in the subacute-to-chronic recovery phase at time of transfer), including baseline data collection, fiberoptic laryngoscopy, and swallowing function evaluation, to comply with the ‘simultaneous measurement’ design principle of cross-sectional studies. It should be noted that, given the specialized neurological rehabilitation setting of this study, the majority of patients were in the subacute-to-chronic recovery phase at the time of enrollment, as reflected by the median disease duration of 77.0 days (IQR: 40.0–179.5) in the VFMI group and 89.5 days (IQR: 42.8–175.3) in the non-VFMI group; the 48–72 h window refers to the time since admission to our center rather than since initial disease onset. For patients classified as minimally conscious, the FEES examination focused on endoscopic observation of vocal fold position, mobility, and secretion status in the resting state; formal bolus swallowing challenge tasks were only administered when patients demonstrated sufficient responsiveness and intact protective reflexes to ensure procedural safety. Patients in coma who could not be safely assessed were excluded during the screening phase as part of the incomplete data exclusion criteria. Based on laryngoscopy results, patients were categorized into the non-VFMI group (*n* = 60, 21.4%) and the VFMI group (*n* = 220, 78.6%).

### 2.2 Data collection and variable definitions

The primary outcome measure was the presence or absence of VFMI, defined as a binary variable: 0 representing normal vocal fold movement and 1 representing VFMI. VFMI was defined endoscopically as complete fixation (no visible movement) or hypomotility (visibly reduced but present movement) of one or both vocal folds. Vocal fold motor function was assessed through fiberoptic laryngoscopy, performed by a professionally trained swallowing rehabilitation physician with 20 years of clinical experience. Patients were positioned in a sitting or semi-recumbent position. After topical nasal anesthesia, a fiberoptic laryngoscope (ATMOS Scope, Germany) was inserted through the nasal cavity to the pharyngolaryngeal region for direct visualization of vocal fold position, symmetry, and mobility. The examination process was video-recorded and archived, with blinded interpretation performed by two independent evaluators; discrepancies were resolved through consensus discussion. It is important to emphasize that laryngeal electromyography (L-EMG) was not performed in this study. Accordingly, the term ‘vocal fold motion impairment (VFMI)’ is used throughout this manuscript as a purely descriptive, endoscopy-based phenotypic classification of vocal fold motor function, and does not imply confirmed neurogenic etiology or electrophysiologically verified denervation. The possibility that some cases classified as hypomotility reflect mechanical causes (e.g., arytenoid dislocation, cricoarytenoid joint restriction) or that some cases of apparent complete immobility represent non-neurogenic fixation cannot be excluded. VFMI was further subclassified by severity as complete vocal fold immobility (no visible movement) or vocal fold hypomotility (visibly reduced but present movement), and by laterality as unilateral or bilateral. Normal vocal fold movement was defined as the absence of VFMI. Among patients with VFMI (*n* = 220), subgroup distribution by laterality and severity is presented in Table 1. For exploratory subgroup-stratified regression analyses, the primary outcome was further operationalized as two binary dependent variables: laterality (0 = unilateral VFMI; 1 = bilateral VFMI) and severity (0 = hypomotility; 1 = complete vocal fold immobility), as described in Section 2.3 and reported in Section 3.4.

**Table 1:** Comparison of baseline characteristics between two groups.

Characteristic	Non-VFMI group (*n* = 60)	VFMI group (*n* = 220)	Statistic	*p*-value
Age (years), *M* (*Q*1, *Q*3)	52.5 (39.5, 60.0)	55.0 (44.0, 61.0)	*W* = 5839.5	0.172
Gender, *n* (%)			*χ*^2^ = 1.23	0.267
Male	49 (81.7)	162 (73.6)		
Female	11 (18.3)	58 (26.4)		
Primary diagnosis, *n* (%)			*χ*^2^ = 4.79	0.323
Cerebral hemorrhage	28 (46.7)	79 (35.9)		
Cerebral infarction	13 (21.7)	51 (23.2)		
Traumatic brain injury	13 (21.7)	44 (20.0)		
Brain tumor	3 (5.0)	17 (7.7)		
Others	3 (5.0)	29 (13.2)		
Lesion location, *n* (%)			*χ*^2^ = 0.60	0.742
Supratentorial	34 (56.7)	118 (53.6)		
Infratentorial	11 (18.3)	36 (16.4)		
Others	15 (25.0)	66 (30.0)		
Disease duration (days), *M* (*Q*1, *Q*3)	77.0 (40.0, 179.5)	89.5 (42.8, 175.3)	*W* = 5971.5	0.259
Consciousness level, *n* (%)			*W* = 6,354	0.611
Alert	40 (66.7)	132 (60.0)		
Minimally conscious	4 (6.7)	37 (16.8)		
Coma	16 (26.7)	51 (23.2)		
Tracheostomy, *n* (%)			*χ*^2^ = 0.87	0.351
Yes	16 (26.7)	75 (34.1)		
No	44 (73.3)	145 (65.9)		
Comorbidities, *n* (%)				
Hypertension	37 (61.7)	107 (48.6)	*χ*^2^ = 2.70	0.100
Diabetes	11 (18.3)	41 (18.6)	*χ*^2^ < 0.01	1.000
Hyperlipidemia	0 (0.0)	1 (0.5)	*χ*^2^ < 0.01	1.000
Pneumonia	8 (13.3)	53 (24.1)	*χ*^2^ = 2.60	0.107

Data are presented as *n* (%) or median (interquartile range). *M* = median; *Q*1, *Q*3 = first and third quartiles; *W* = Wilcoxon rank-sum statistic; *χ*^2^ = chi-square statistic.

Independent variables included a total of 32 variables encompassing demographic characteristics, comorbidities, and swallowing function-related indicators. Swallowing function was comprehensively assessed through standardized bedside swallowing evaluation and fiberoptic endoscopic evaluation of swallowing (FEES), covering 18 dimensional indicators classified into 5 categories: (1) Secretion management capacity (nasopharyngeal secretions, vallecula secretions, left pyriform sinus secretions, right pyriform sinus secretions, laryngeal vestibule secretions, using 5-point grading: Grade 0 = normal/no secretions, Grade 1 = minimal/thin, Grade 2 = mild/moderate amount or slightly viscous, Grade 3 = moderate/large amount or significantly viscous, Grade 4 = severe/filled or extremely viscous with severe airway obstruction); (2) Swallowing physiology mechanisms (velopharyngeal closure function, tongue base retraction function, pharyngeal wall movement function, using 3-point grading: 1 = normal, 2 = reduced, 3 = absent/unable); (3) Protective reflex mechanisms (pharyngeal reflex, cough reflex, cough effectiveness, using 3-point grading); (4) Residue severity assessment (vallecula residue, pyriform sinus residue, using 5-point grading: Grade 0 = no residue, Grade 1 = minimal <25% filling, Grade 2 = mild 25–50%, Grade 3 = moderate 50–75%, Grade 4 = severe >75% filling); (5) Aspiration risk assessment (Rosenbek Penetration-Aspiration Scale, 8 grades); and other indicators (Murray secretion scale, swallowing frequency, presence or absence of local swelling, nutritional method).

Covariates included available demographic and clinical characteristics: age, sex, primary diagnosis type, lesion location, disease duration, level of consciousness (categorized into three tiers: alert, minimally conscious, or coma, based on bedside clinical assessment conducted on the day of FEES evaluation), tracheostomy status, and major comorbidities including hypertension, diabetes, hyperlipidemia, and pneumonia. Detailed endotracheal intubation history and standardized neurological severity scores such as GCS or NIHSS were not consistently available for all participants and therefore could not be incorporated into the multivariable model. All data were collected synchronously on the day of assessment.

### 2.3 Statistical analysis methods

All statistical analyses were performed using R software (version 4.3.3), with a significance level set at *α* = 0.05 (two-tailed test). Continuous variables conforming to normal distribution and homogeneity of variance were expressed as mean ± standard deviation; otherwise, median (interquartile range) was used. Categorical variables were expressed as frequencies (percentages).

Univariable analysis for between-group comparisons: Categorical variables were analyzed using chi-square test or Fisher’s exact test (depending on whether expected frequencies in each cell were ≥5); continuous variables, confirmed by Shapiro–Wilk test to be non-normally distributed, were analyzed using Mann–Whitney *U* test. All tests reported corresponding statistics [Wilcoxon rank-sum statistic (*W*) or chi-square statistic (*χ*^2^)].

Multivariable logistic regression analysis: Eighteen variables with *p* < 0.05 in univariable analysis were included in the preliminary model. To avoid overfitting and improve model stability, ordinal categorical variables were treated as continuous variables. Variance inflation factor (VIF) was used to detect multicollinearity, with VIF > 10 set as the exclusion criterion. When two indicators exhibited extreme collinearity (Spearman *r* > 0.9), a composite maximum-score variable was constructed to avoid arbitrary single-side selection. Accordingly, the bilateral pyriform sinus secretion pooling variables (Spearman *r* = 0.974, *p* < 0.001) were combined into PS_MAX = max[left score, right score] as the primary exposure variable. A stepwise iterative method was employed to eliminate remaining collinear variables, ultimately retaining 16 variables to construct the multivariable logistic regression model. Regression coefficients (*β*), standard errors, Wald statistics, *p*-values, odds ratios (OR), and 95% confidence intervals were calculated for each variable.

The following R packages were used for multivariable analysis: tidyverse, car, broom, jtools, pROC, rms, ResourceSelection, DescTools, ggpubr, cowplot, showtext. The pROC package was used to construct the receiver operating characteristic (ROC) curve and calculate the area under the curve (AUC) with 95% confidence intervals to assess the discriminatory performance of the final multivariable model. The optimal cutoff point was determined using the Youden index (sensitivity + specificity − 1). Forest plots of adjusted odds ratios and 95% confidence intervals were generated using ggplot2 within the tidyverse framework.

### 2.4 Ethics and informed consent

This study protocol was approved by the Medical Ethics Committee of Guangdong Sanjiu Brain Hospital (Ethics approval number: 2024-01-034, approval date: June 12, 2024) and strictly adhered to the ethical principles of the Declaration of Helsinki. All enrolled patients or their legal guardians signed written informed consent after fully understanding the research objectives, procedures, potential risks, and benefits. Patients had the right to withdraw from the study unconditionally at any stage without affecting their normal medical care. All patient data were anonymized and used solely for scientific research purposes.

## 3 Results

### 3.1 Comparison of baseline characteristics

This study included a total of 280 patients with brain injury, who were categorized into the non-VFMI group with 60 cases (21.4%) and the VFMI group with 220 cases (78.6%) based on fiberoptic endoscopic assessment of vocal cord movement. No statistically significant differences were observed between the two groups in baseline characteristics including demographic features, disease type, lesion location, disease duration, level of consciousness, tracheostomy status, and major comorbidities (all *p* > 0.05, Table 1), indicating good comparability between groups.

### 3.2 Univariable analysis of swallowing function indicators

Univariable analysis revealed that 18 FEES-based swallowing function indicators showed statistically significant differences between the non-VFMI group and the VFMI group (all *p* < 0.05, Table 2). These indicators encompassed multiple dimensions including secretion management, swallowing physiology mechanisms, protective reflexes, residue severity, and aspiration risk.

**Table 2:** Univariate comparison of swallowing function indicators between two groups.

Indicator	Non-VFMI group (*n* = 60)	VFMI group (*n* = 220)	Statistic	*p*-value
Nasopharyngeal secretions, *n* (%)			*W* = 5,289	<0.001
Normal	14 (23.33)	17 (7.73)		
Trace	44 (73.33)	182 (82.73)		
Mild	2 (3.33)	20 (9.09)		
Moderate	0 (0.00)	1 (0.45)		
Severe	0 (0.00)	0 (0.00)		
Vallecula secretions, *n* (%)			*W* = 3740.5	<0.001
Normal	22 (36.67)	15 (6.82)		
Trace	26 (43.33)	95 (43.18)		
Mild	8 (13.33)	63 (28.64)		
Moderate	1 (1.67)	28 (12.73)		
Severe	3 (5.00)	19 (8.64)		
Pyriform sinus secretions (left), *n* (%)			*W* = 3432.5	<0.001
Normal	18 (30.00)	15 (6.82)		
Trace	27 (45.00)	53 (24.09)		
Mild	8 (13.33)	79 (35.91)		
Moderate	4 (6.67)	51 (23.18)		
Severe	3 (5.00)	22 (10.00)		
Pyriform sinus secretions (right), *n* (%)			*W* = 3377.5	<0.001
Normal	18 (30.00)	14 (6.36)		
Trace	27 (45.00)	52 (23.64)		
Mild	8 (13.33)	82 (37.27)		
Moderate	4 (6.67)	49 (22.27)		
Severe	3 (5.00)	23 (10.45)		
Pyriform sinus secretions (MAX), *n* (%)			*W* = 3365.5	<0.001
Normal	18 (30.00)	14(6.36364%)		
Trace	27 (45.00)	52(23.63636%)		
Mild	8 (13.33)	80(36.36364%)		
Moderate	4 (6.67)	51(23.18182%)		
Severe	3 (5.00)	23(10.45455%)		
Laryngeal vestibule secretions, *n* (%)			*W* = 4,529	<0.001
Normal	49 (81.67)	112 (50.91)		
Trace	7 (11.67)	58 (26.36)		
Mild	2 (3.33)	26 (11.82)		
Moderate	0 (0.00)	16 (7.27)		
Severe	2 (3.33)	8 (3.64)		
Murray scale, *n* (%)			*W* = 4337.5	<0.001
Grade 0	48 (80.00)	101 (45.91)		
Grade 1	5 (8.33)	50 (22.73)		
Grade 2	5 (8.33)	45 (20.45)		
Grade 3	2 (3.33)	24 (10.91)		
Swallowing frequency, *n* (%)			*W* = 4,798	<0.001
≥3 times/min	40 (66.67)	88 (40.00)		
<3 times/min	17 (28.33)	108 (49.09)		
No spontaneous swallow	3 (5.00)	24 (10.91)		
Velopharyngeal closure, *n* (%)			*W* = 5,469	0.001
Normal	12 (20.00)	13 (5.91)		
Incomplete	46 (76.67)	190 (86.36)		
Unable	2 (3.33)	17 (7.73)		
Tongue base retraction, *n* (%)			*W* = 5,603	0.004
Normal	8 (13.33)	9 (4.09)		
Reduced	50 (83.33)	188 (85.45)		
Absent	2 (3.33)	23 (10.45)		
Pharyngeal wall movement, *n* (%)			*W* = 4,754	<0.001
Normal	13 (21.67)	0 (0.00)		
Reduced	26 (43.33)	104 (47.27)		
Absent	21 (35.00)	116 (52.73)		
Pharyngeal reflex (sensory), *n* (%)			*W* = 5,052	<0.001
Normal	21 (35.00)	34 (15.45)		
Weak response	38 (63.33)	168 (76.36)		
No response	1 (1.67)	18 (8.18)		
Cough reflex, *n* (%)			*W* = 5,342	0.005
Normal	25 (41.67)	51 (23.18)		
Delayed	34 (56.67)	162 (73.64)		
Absent	1 (1.67)	7 (3.18)		
Cough effectiveness, *n* (%)			*W* = 4261.5	<0.001
Complete	3 (5.00)	0 (0.00)		
Somewhat	24 (40.00)	35 (15.91)		
Slightly effective	30 (50.00)	146 (66.36)		
Ineffective	3 (5.00)	39 (17.73)		
Vallecula residue grading, *n* (%)			*W* = 4424.5	<0.001
None	12 (20.00)	9 (4.09)		
Trace	21 (35.00)	64 (29.09)		
Mild	20 (33.33)	76 (34.55)		
Moderate	4 (6.67)	40 (18.18)		
Severe	3 (5.00)	31 (14.09)		
Pyriform sinus residue grading, *n* (%)			*W* = 4260.5	<0.001
None	7 (11.67)	5 (2.27)		
Trace	28 (46.67)	57 (25.91)		
Mild	13 (21.67)	64 (29.09)		
Moderate	7 (11.67)	56 (25.45)		
Severe	5 (8.33)	38 (17.27)		
Rosenbek Penetration-Aspiration Scale, *n* (%)			*W* = 3638.5	<0.001
Grade 1	24 (40.00)	23 (10.45)		
Grade 2	8 (13.33)	28 (12.73)		
Grade 3	1 (1.67)	2 (0.91)		
Grade 4	17 (28.33)	53 (24.09)		
Grade 5	3 (5.00)	30 (13.64)		
Grade 6	1 (1.67)	24 (10.91)		
Grade 7	3 (5.00)	34 (15.45)		
Grade 8	3 (5.00)	26 (11.82)		
Local swelling, *n* (%)			*χ*^2^ = 5.885	0.015
Present	4 (6.67)	47 (21.36)		
Absent	56 (93.33)	173 (78.64)		
Nutrition method, *n* (%)			*χ*^2^ = 7.359	0.007
Tube feeding	39 (65.00)	181 (82.27)		
Oral intake	21 (35.00)	39 (17.73)		

Data are presented as *n* (%). *W* = Wilcoxon rank-sum statistic; *χ*^2^ = chi-square statistic. Ordinal categorical variables were analyzed using Mann–Whitney *U* test; binary and unordered multicategorical variables were analyzed using chi-square test or Fisher’s exact test. *p* < 0.05 indicates statistically significant difference.

In the secretion management dimension, the severity of secretions in the nasopharynx, vallecula, bilateral pyriform sinuses, and laryngeal vestibule all showed significant differences between the two groups (all *p* < 0.001). Notably, patients in the VFMI group exhibited markedly higher degrees of pyriform sinus secretion pooling compared to the non-VFMI group, with a significantly increased proportion of mild or greater secretion pooling. The Murray secretion scale also demonstrated a higher proportion of high-grade secretions in the VFMI group.

Regarding swallowing physiology mechanisms, pharyngeal wall movement, velopharyngeal closure function, tongue base retraction ability, and swallowing frequency showed significant differences between groups. In the VFMI group, the proportion of patients with reduced or absent pharyngeal wall movement was markedly elevated, with not a single patient demonstrating completely normal pharyngeal wall movement.

Among airway protection-related indicators, pharyngeal reflex, cough reflex, and cough effectiveness were all significantly impaired in the VFMI group (*p* ≤ 0.005), suggesting that abnormal vocal cord movement is frequently accompanied by reduced airway protective reflex function.

In terms of residue assessment, both vallecula residue and pyriform sinus residue evaluated using the Yale grading method showed significant differences between groups (all *p* < 0.001), with the VFMI group demonstrating a markedly elevated proportion of moderate-to-severe residue.

Additionally, the Rosenbek Penetration-Aspiration Scale revealed that the proportion of high-grade aspiration (≥Grade 4) in the VFMI group was significantly higher than that in the non-VFMI group, with a notably increased proportion of silent aspiration.

### 3.3 Multivariable logistic regression analysis

Based on univariable analysis, swallowing function indicators and clinically relevant covariates with statistical significance were included in the multivariable logistic regression model. After detecting and addressing multicollinearity using variance inflation factor (VIF), the final model included 16 variables. It is important to note that both left and right pyriform sinus secretion pooling were extremely significantly associated with VFMI in univariable analysis (left side: Mann–Whitney *U* test, *p* = 3.97 × 10^−9^, Cliff’s Delta = −0.48, 95% CI: −0.61 to −0.32; right side: Mann–Whitney *U* test, *p* = 2.07 × 10^−9^, Cliff’s Delta = −0.49, 95% CI: −0.62 to −0.33). The effect sizes for both sides were nearly identical (rank-biserial correlation coefficient: left side 0.48, right side 0.49), and Spearman correlation analysis revealed an extremely high correlation between the two (*r* = 0.974, *p* < 0.001), indicating 94.8% shared variance with nearly complete clinical concordance between bilateral manifestations. In multivariable regression analysis, this extremely high correlation (*r* > 0.9) would lead to severe collinearity problems, rendering regression coefficient estimates highly unstable (details available in Supplementary materials). To avoid arbitrary single-side selection, we constructed a composite maximum-score variable [PS_MAX = max(left score, right score)] as the primary exposure in the multivariable model. PS_MAX was a significant independent co-manifestation indicator of VFMI (OR = 2.165, 95% CI: 1.067–4.396, *p* = 0.032).

**Figure 1:**
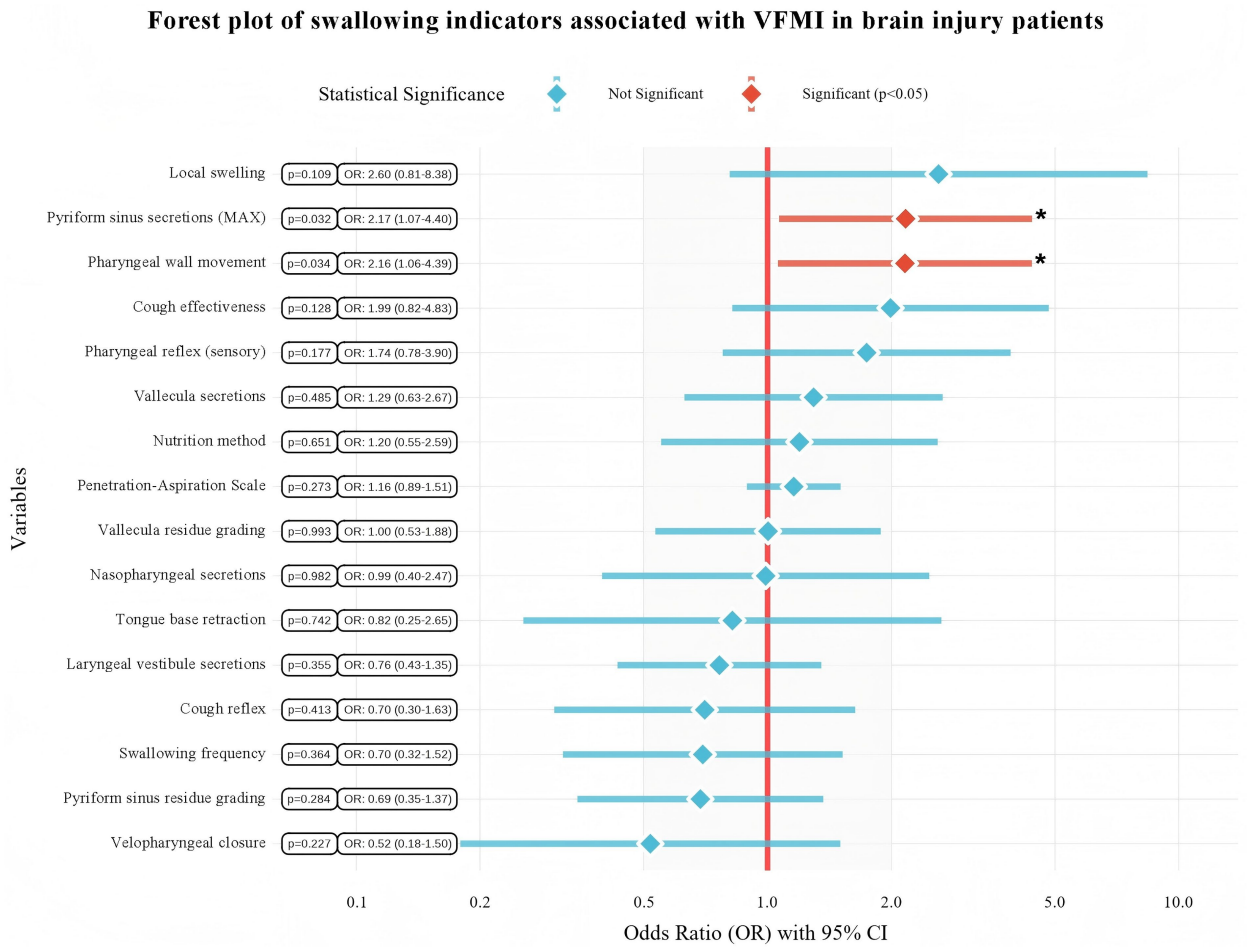
Forest plot of the multivariable logistic regression model: adjusted odds ratios (ORs) and 95% confidence intervals (CIs) for swallowing function indicators and their co-manifestation with vocal fold motion impairment (VFMI) in patients with brain injury. Red diamonds (★) indicate statistically significant variables (*P* < 0.05); blue diamonds indicate non-significant variables. The vertical dashed red line represents OR = 1.0 (null effect). Model includes the composite pyriform sinus secretion variable (PS_MAX = max[left, right]).

**Figure 2:**
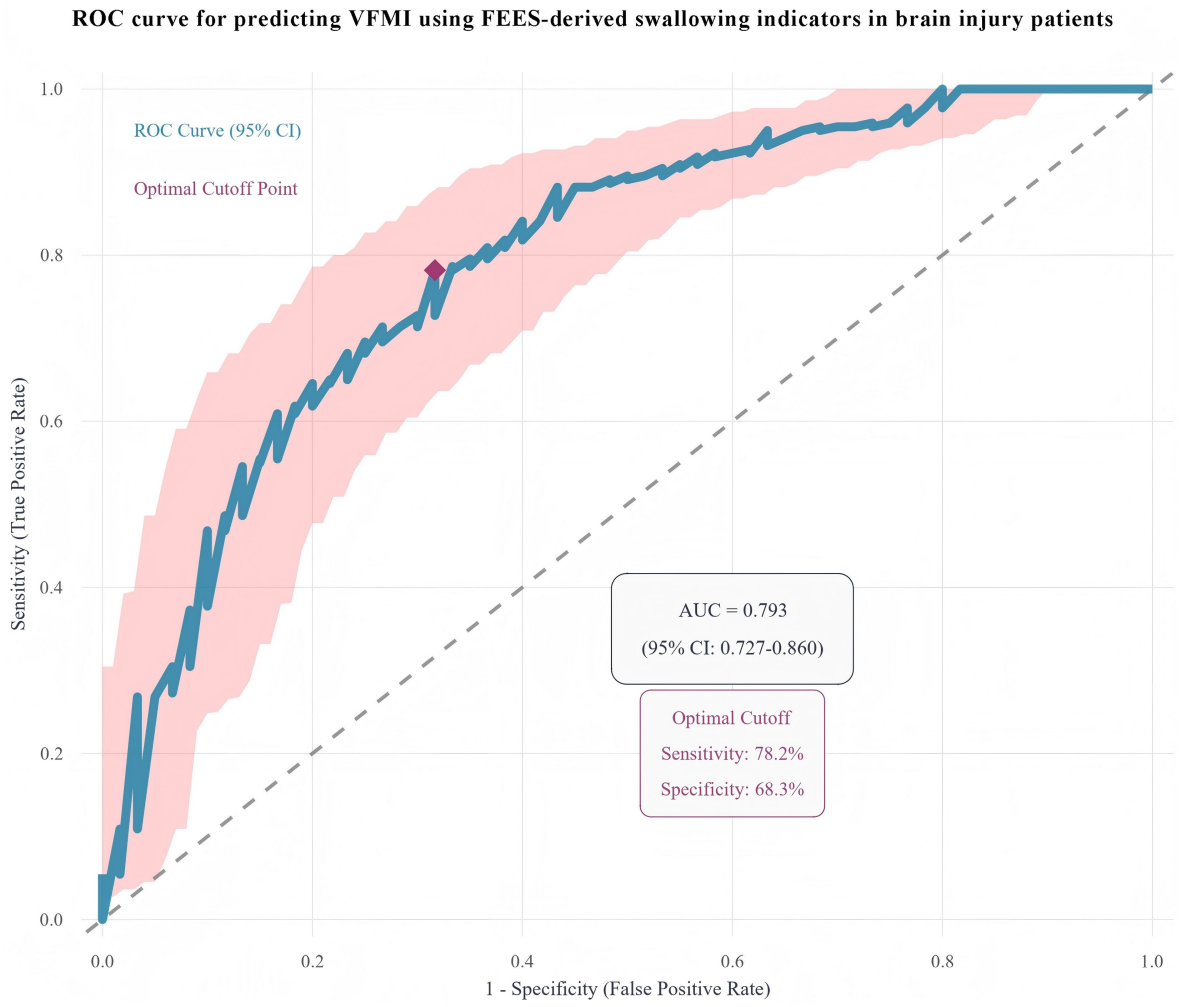
Receiver operating characteristic (ROC) curve for the multivariable logistic regression model predicting co-manifestation of vocal fold motion impairment (VFMI) based on FEES-derived swallowing function indicators in patients with brain injury. AUC = 0.793 (95% CI: 0.727–0.860). The pink shading represents the 95% confidence band. The red diamond indicates the optimal cutoff point determined by the Youden index (sensitivity = 78.2%, specificity = 68.3%).

Multivariable analysis results demonstrated that after adjusting for available clinical confounders, including age, sex, diagnosis type, lesion location, disease duration, categorical level of consciousness, tracheostomy status, and major comorbidities, restricted pharyngeal wall movement (OR = 2.158, 95% CI: 1.060–4.392, *p* = 0.034) and pyriform sinus secretion (PS_MAX: OR = 2.165, 95% CI: 1.067–4.396, *p* = 0.032) showed significant and consistent co-manifestation with VFMI, reflecting their shared pharyngolaryngeal neural substrate (Table 3). The complete multivariable logistic regression results for all 16 variables—including regression coefficients, standard errors, Wald statistics, odds ratios, 95% confidence intervals, and *p*-values—are visualized in Figure 1 (Forest plot). The two statistically significant variables—restricted pharyngeal wall movement (OR = 2.158, 95% CI: 1.060–4.392, *p* = 0.034) and pyriform sinus secretion pooling (PS_MAX: OR = 2.165, 95% CI: 1.067–4.396, *p* = 0.032)—are highlighted in Figure 1. The overall discriminatory performance of the multivariable model was assessed using ROC curve analysis (Figure 2). The model achieved an AUC of 0.793 (95% CI: 0.727–0.860), indicating good discrimination between patients with and without VFMI. At the optimal Youden cutoff (threshold = 0.676), sensitivity was 78.2% and specificity was 68.3%. Model calibration was confirmed by the Hosmer-Lemeshow test (*p* = 0.596 > 0.05).

**Table 3:** Multivariate logistic regression analysis for vocal fold motion impairment (final model).

Variable	*β*	SE	Wald *χ*^2^	OR	95% CI	*p*-value
Pyriform sinus secretions (MAX)	0.773	0.361	4.574	2.165	1.067–4.396	0.032*
Pharyngeal wall movement	0.769	0.363	4.497	2.158	1.060–4.392	0.034*
Local swelling	0.952	0.596	2.548	2.591	0.805–8.338	0.110
Cough effectiveness	0.684	0.452	2.292	1.982	0.817–4.805	0.130
Pharyngeal reflex (sensory)	0.559	0.411	1.847	1.748	0.781–3.914	0.174
Velopharyngeal closure	−0.654	0.543	1.453	0.520	0.179–1.506	0.228
Rosenbek Penetration-Aspiration Scale	0.148	0.134	1.224	1.160	0.892–1.508	0.269
Pyriform sinus residue grading	−0.365	0.350	1.089	0.694	0.349–1.378	0.297
Laryngeal vestibule secretions	−0.268	0.292	0.843	0.765	0.432–1.355	0.358
Swallowing frequency	−0.355	0.399	0.789	0.701	0.321–1.534	0.374
Cough reflex	−0.347	0.430	0.654	0.707	0.305–1.640	0.419
Vallecula secretions	0.279	0.368	0.577	1.322	0.643–2.718	0.448
Nutrition method	0.168	0.394	0.181	1.183	0.546–2.560	0.670
Tongue base retraction	−0.202	0.597	0.115	0.817	0.253–2.632	0.735
Vallecula residue grading	−0.006	0.321	<0.001	0.994	0.529–1.867	0.985
Nasopharyngeal secretions	−0.004	0.468	<0.001	0.996	0.398–2.491	0.993

*β*, regression coefficient; SE, standard error; Wald *χ*^2^, Wald chi-square statistic; OR, odds ratio; CI, confidence interval. Model goodness-of-fit: Hosmer–Lemeshow test *p* = 0.596 (*p* > 0.05 suggests good model fit). Multicollinearity detection: all variables VIF<5.0. ^*^p < 0.05 indicates statistically significant difference.

### 3.4 Exploratory subgroup analyses: swallowing Indicator associations by VFMI laterality and severity

To further characterize the functional heterogeneity within the VFMI subpopulation, two exploratory subgroup-stratified multivariable logistic regression analyses were conducted among the 220 patients with confirmed VFMI, examining swallowing indicator associations by laterality (unilateral vs. bilateral) and severity (hypomotility vs. complete immobility) separately. These within-VFMI subtype comparisons were performed to avoid logical redundancy in reference group assignment that would arise from comparing each subtype against the non-VFMI group. All analyses were adjusted for age, sex, diagnosis type, lesion location, disease duration, level of consciousness, tracheostomy status, and major comorbidities. VIF screening confirmed no variable exceeded the exclusion threshold (VIF > 10) in either model.

#### 3.4.1 Subgroup analysis by laterality: unilateral vs. bilateral VFMI

Among the 220 patients with VFMI, laterality subtyping was derived from four vocal fold movement variables (left and right adduction and abduction): left-side abnormality was defined as any score ≠ 1 (normal) on left adduction or left abduction; right-side abnormality was defined similarly. Bilateral VFMI (dependent variable coded 1) was defined as abnormalities present on both sides; unilateral VFMI (coded 0) as abnormality on one side only.

**Figure 3:**
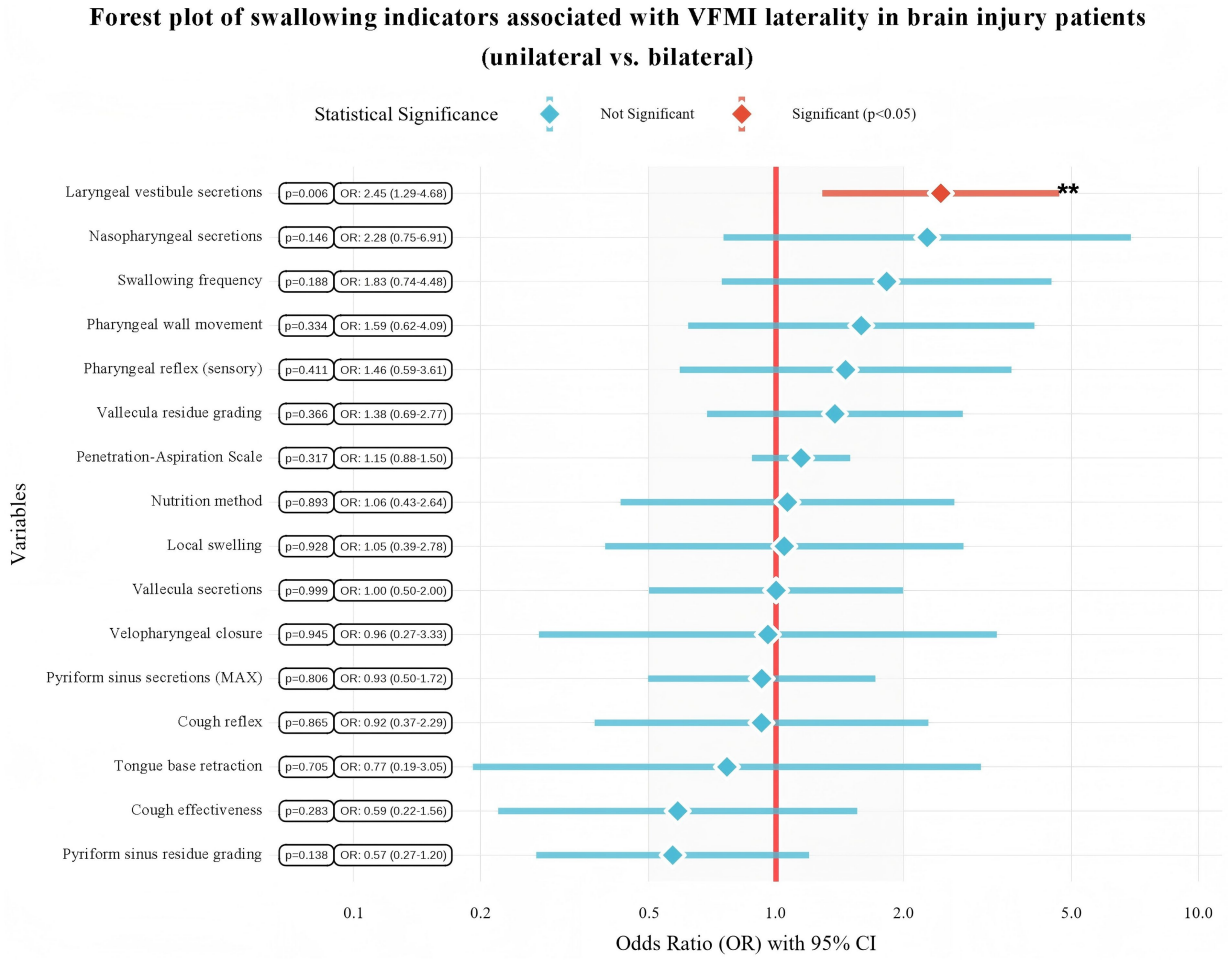
Multivariable logistic regression results (n = 220 patients with VFMI) examining swallowing function indicators as predictors of VFMI laterality, comparing bilateral VFMI (coded 1) against unilateral VFMI (coded 0). All analyses were adjusted for age, sex, diagnosis type, lesion location, disease duration, level of consciousness, tracheostomy status, and major comorbidities. Odds ratios (OR) and 95% confidence intervals (CI) are plotted on a logarithmic scale. Diamond markers indicate point estimates: red/orange diamonds denote statistically significant associations (p < 0.05); teal diamonds denote non-significant associations. Laryngeal vestibule secretion accumulation was the sole indicator independently associated with bilateral VFMI (OR = 2.45, 95% CI: 1.29–4.68, *p* = 0.006).

**Figure 4:**
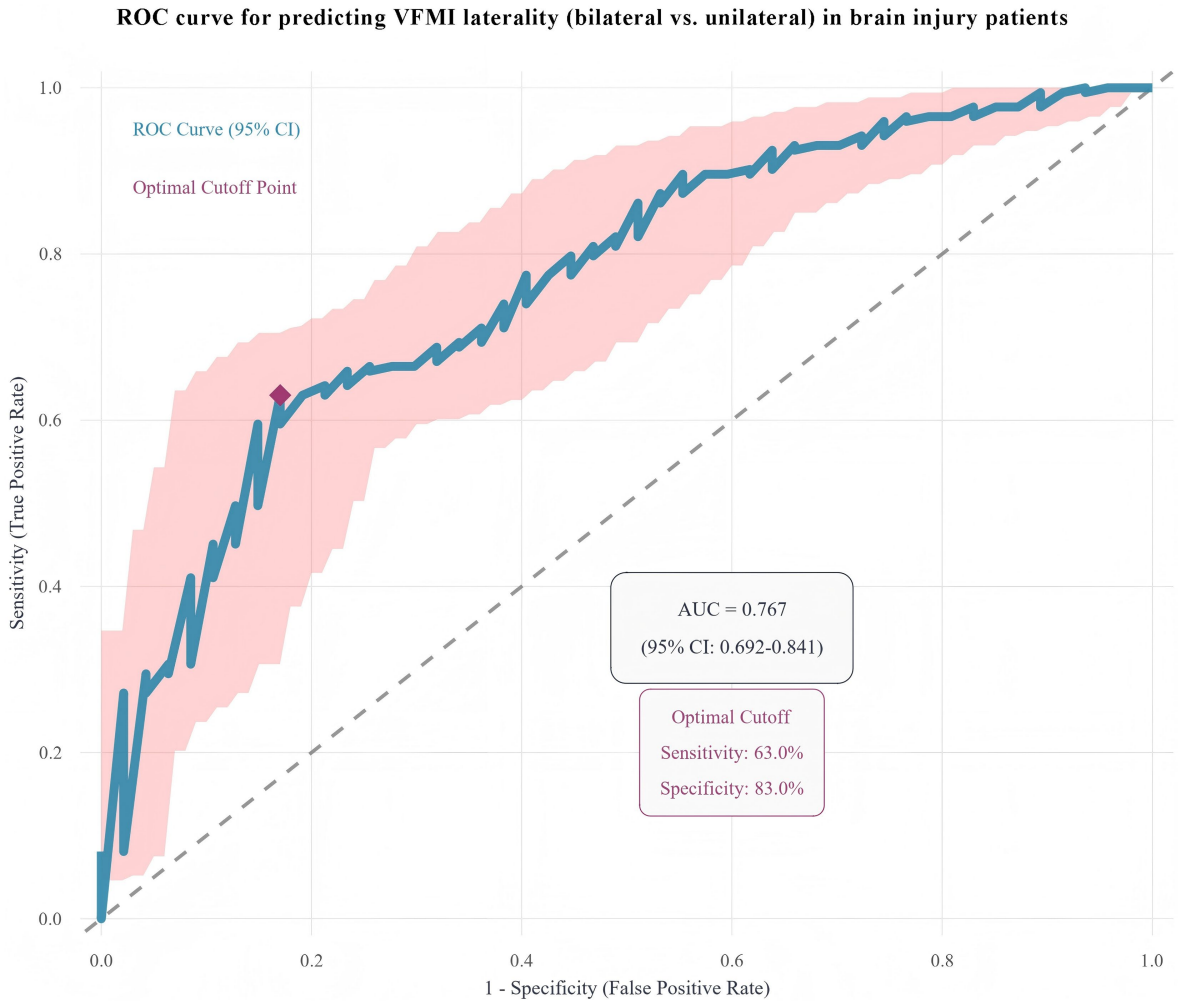
Receiver operating characteristic (ROC) curve illustrating the discriminatory performance of the multivariable logistic regression model for distinguishing bilateral from unilateral VFMI among 220 patients with VFMI. The shaded pink region represents the 95% confidence interval (CI) of the ROC curve. The area under the curve (AUC) was 0.767 (95% CI: 0.692–0.841), indicating good discriminatory performance. The optimal cutoff point (pink diamond marker), determined by the Youden index, corresponded to a sensitivity of 63.0% and specificity of 83.0%.

After multivariable adjustment, laryngeal vestibule secretion accumulation was the sole swallowing indicator that independently distinguished bilateral from unilateral VFMI (*β* = 0.897, OR = 2.454, 95% CI: 1.287–4.680, *p* = 0.006). No other swallowing indicator among the remaining 15 variables reached statistical significance (all *p* > 0.05). Complete multivariable results for all 16 variables are presented in Table 4. The model demonstrated good discriminatory performance, with an AUC of 0.767 (95% CI: 0.692–0.841). Model calibration was satisfactory as confirmed by the Hosmer–Lemeshow goodness-of-fit test (*p* = 0.219). At the optimal Youden cutoff (threshold = 0.804), sensitivity was 63.0% and specificity was 83.0%(details available in Figures 3–4).

**Table 4:** Exploratory subgroup multivariable logistic regression analysis by VFMI laterality: unilateral vs. bilateral vocal fold motion impairment (*n* = 220).

Variable	*β*	SE	Wald *χ*^2^	OR	95% CI	*p* value
Laryngeal vestibule secretions	0.897	0.325	7.610	2.454	1.287–4.680	0.006*
Swelling	0.712	0.598	1.419	2.038	0.631–6.583	0.234
Pyriform sinus secretions (PS_MAX)	0.341	0.287	1.413	1.406	0.802–2.466	0.234
Pharyngeal wall movement	0.288	0.312	0.852	1.334	0.724–2.459	0.356
Cough effectiveness	0.261	0.441	0.350	1.298	0.547–3.081	0.554
Gag reflex	0.197	0.392	0.253	1.218	0.565–2.626	0.615
Nutrition	0.163	0.388	0.177	1.177	0.550–2.519	0.674
Rosenbek PAS	0.122	0.136	0.804	1.130	0.866–1.474	0.370
Vallecula secretions	0.091	0.362	0.063	1.095	0.539–2.225	0.802
Nasopharyngeal secretions	−0.092	0.453	0.041	0.912	0.375–2.215	0.840
Vallecula residue rating	−0.118	0.323	0.134	0.889	0.472–1.673	0.714
Tongue base retraction	−0.159	0.596	0.071	0.853	0.265–2.742	0.790
Cough reflex	−0.174	0.414	0.177	0.840	0.373–1.894	0.674
Swallowing frequency	−0.201	0.389	0.267	0.818	0.381–1.755	0.605
Piriform sinus residue rating	−0.213	0.341	0.390	0.808	0.413–1.580	0.532
Velopharyngeal closure	−0.498	0.548	0.825	0.608	0.207–1.783	0.364

Laterality coding derived from four vocal fold movement variables (adduction and abduction, left and right): left-side abnormality defined as any score ≠ 1 (normal) on left adduction or left abduction; right-side abnormality defined similarly. Bilateral VFMI (coded 1) = abnormalities present on both sides; unilateral VFMI (coded 0) = abnormality on one side only. All analyses adjusted for age, sex, diagnosis type, lesion location, disease duration, level of consciousness, tracheostomy status, and major comorbidities. Results are exploratory and hypothesis-generating; VIF screening confirmed no variable exceeded the exclusion threshold (VIF > 10). ^*^p < 0.05 indicates statistically significant difference.

#### 3.4.2 Subgroup analysis by severity: hypomotility vs. complete vocal fold immobility

Severity subtyping was derived from the peak score across the four vocal fold movement variables (left and right adduction and abduction): a peak score of 2 (visibly reduced but present movement) was classified as hypomotility (coded 0), and a peak score of 3 (no visible movement) was classified as complete vocal fold immobility (coded 1).

**Figure 5:**
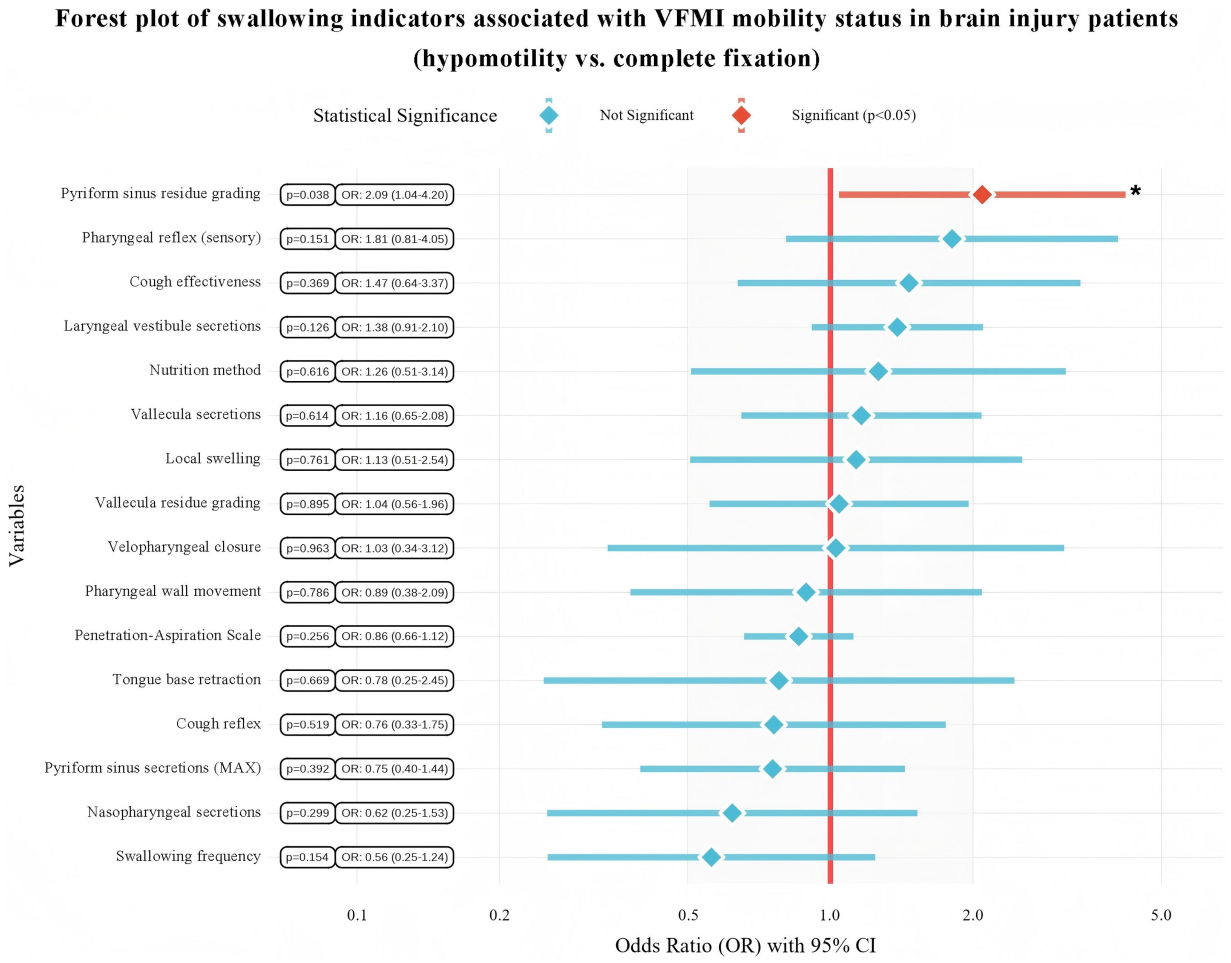
Multivariable logistic regression results (n = 220 patients with VFMI) examining swallowing function indicators as predictors of VFMI severity, comparing complete vocal fold immobility (coded 1) against hypomotility (coded 0). All analyses were adjusted for age, sex, diagnosis type, lesion location, disease duration, level of consciousness, tracheostomy status, and major comorbidities. Odds ratios (OR) and 95% confidence intervals (CI) are plotted on a logarithmic scale. Diamond markers indicate point estimates: red/orange diamonds denote statistically significant associations (p < 0.05); teal diamonds denote non-significant associations. Pyriform sinus residue grading was the sole indicator independently associated with complete vocal fold immobility (OR = 2.09, 95% CI: 1.04–4.20, *p* = 0.038).

**Figure 6:**
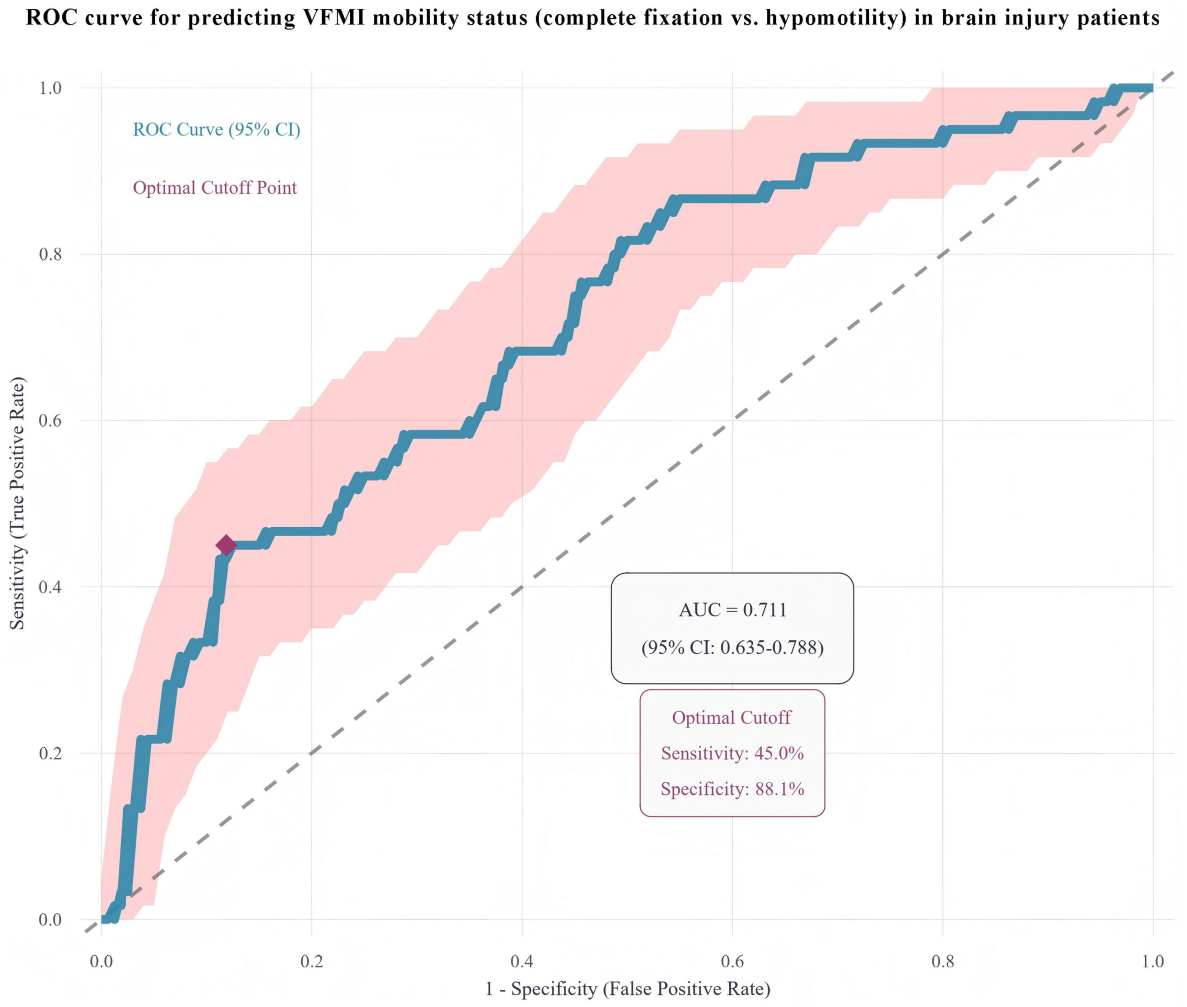
Receiver operating characteristic (ROC) curve illustrating the discriminatory performance of the multivariable logistic regression model for distinguishing complete vocal fold immobility from hypomotility among 220 patients with VFMI. The shaded pink region represents the 95% confidence interval (CI) of the ROC curve. The area under the curve (AUC) was 0.711 (95% CI: 0.635–0.788), indicating moderate discriminatory performance. The optimal cutoff point (pink diamond marker), determined by the Youden index, corresponded to a sensitivity of 45.0% and specificity of 88.1%.

After multivariable adjustment, pyriform sinus residue rating was the sole swallowing indicator that independently distinguished complete vocal fold immobility from hypomotility (*β* = 0.738, OR = 2.093, 95% CI: 1.042–4.202, *p* = 0.038). No other swallowing indicator among the remaining 15 variables reached statistical significance (all *p* > 0.05). Complete multivariable results for all 16 variables are presented in Table 5. The model demonstrated moderate discriminatory performance, with an AUC of 0.711 (95% CI: 0.635–0.788). Model calibration was satisfactory as confirmed by the Hosmer-Lemeshow goodness-of-fit test (*p* = 0.368). At the optimal Youden cutoff (threshold = 0.404), sensitivity was 45.0% and specificity was 88.1% (details available in Figures 5–6).

**Table 5:** Exploratory subgroup multivariable logistic regression analysis by VFMI severity: hypomotility vs. complete immobility (*n* = 220).

Variable	*β*	SE	Wald *χ*^2^	OR	95% CI	*p* value
Piriform sinus residue rating	0.738	0.356	4.295	2.093	1.042–4.202	0.038*
Swelling	0.621	0.562	1.224	1.861	0.618–5.603	0.269
Pyriform sinus secretions (PS_MAX)	0.418	0.296	1.992	1.519	0.850–2.714	0.158
Pharyngeal wall movement	0.374	0.326	1.317	1.454	0.767–2.756	0.251
Cough effectiveness	0.312	0.452	0.476	1.366	0.563–3.315	0.490
Rosenbek PAS	0.187	0.143	1.711	1.206	0.910–1.598	0.191
Vallecula secretions	0.154	0.374	0.170	1.167	0.560–2.431	0.680
Gag reflex	0.132	0.403	0.107	1.141	0.518–2.513	0.743
Nutrition	0.108	0.397	0.074	1.114	0.512–2.424	0.786
Nasopharyngeal secretions	0.047	0.467	0.010	1.048	0.420–2.616	0.920
Laryngeal vestibule secretions	−0.063	0.287	0.048	0.939	0.535–1.648	0.826
Vallecula residue rating	−0.098	0.334	0.086	0.907	0.471–1.746	0.769
Tongue base retraction	−0.143	0.612	0.055	0.867	0.261–2.878	0.814
Cough reflex	−0.187	0.427	0.192	0.829	0.359–1.917	0.661
Swallowing frequency	−0.234	0.401	0.341	0.791	0.361–1.735	0.559
Velopharyngeal closure	−0.521	0.563	0.856	0.594	0.197–1.792	0.355

Severity coding derived from the peak score across four vocal fold movement variables (left/right adduction and abduction): peak score = 2 (hypomotility) coded 0; peak score = 3 (complete immobility/fixation) coded 1. All analyses adjusted for age, sex, diagnosis type, lesion location, disease duration, level of consciousness, tracheostomy status, and major comorbidities. Results are exploratory and hypothesis-generating; VIF screening confirmed no variable exceeded the exclusion threshold (VIF > 10). Bold indicates statistical significance (*p* < 0.05). *p < 0.05 indicates statistically significant difference.

## 4 Discussion

Based on a cross-sectional design, this study systematically analyzed the association between multidimensional swallowing function indicators and vocal cord movement status in patients with brain injury through the same fiberoptic endoscopic evaluation platform. Results demonstrated that after adjusting for potential confounding factors including demographic characteristics, disease-related factors, and clinical status, restricted pharyngeal wall movement and pyriform sinus secretion consistently co-manifested with VFMI. These findings confirm that in patients with brain injury, vocal cord movement disorders are not isolated phenomena but co-manifest with pharyngeal phase motor coordination dysfunction and reduced secretion clearance capacity as integrated expressions of shared pharyngolaryngeal neural substrate impairment.

### 4.1 Functional association between dysphagia and VFMI

From the perspective of neuroanatomical and physiological mechanisms, swallowing function and vocal cord movement are highly coupled at the central regulatory level. The brainstem swallowing center, nucleus ambiguus, and their related pathways exhibit significant overlap in spatial distribution and functional integration, with brain injury commonly manifesting as network-level rather than single structural impairment (8, 21, 22). This study found that restricted pharyngeal wall movement consistently co-manifests with VFMI in patients with brain injury, directly reflecting their shared neural injury substrate—specifically, the overlapping brainstem circuits including the nucleus ambiguus and related pathways that co-regulate both pharyngeal constrictor function and vocal cord motor control. This co-manifestation pattern, rather than a mechanistically independent relationship, underscores the integrated nature of pharyngolaryngeal dysfunction following brain injury. While previous studies have predominantly focused on vocal cord function from the perspectives of aspiration or phonation (10, 23, 24), the results of this study further support understanding VFMI within a broader framework of swallowing and airway-protective dysfunction, rather than confining it to either phonatory impairment or swallowing dysfunction in isolation.

The co-manifestation of pyriform sinus secretion pooling and VFMI within a shared neural substrate also holds important functional significance. It is noteworthy that in this study, both left and right pyriform sinus secretion were significantly associated with VFMI in univariable analysis, with an extremely high correlation between the two sides (Spearman *r* = 0.974). In the revised multivariable analysis, a composite maximum-score variable (PS_MAX) formally captured this bilateral information and yielded a significant result (OR = 2.165, *p* = 0.032), reinforcing the conclusion that pyriform sinus secretion pooling co-manifests with VFMI as an expression of shared neural substrate impairment regardless of laterality. Pyriform sinus secretion pooling reflects the combined results of insufficient pharyngeal propulsive force, diminished sensory feedback, and impaired clearance mechanisms (4, 25). In the context of restricted vocal cord movement, incomplete airway closure and reduced secretion clearance capacity often coexist, thereby creating a compounded state of airway protection impairment (26). The findings of this study suggest that secretion pooling is not merely a local phenomenon but may be an external manifestation of overall swallowing-laryngeal functional impairment.

The following subgroup analyses should be considered exploratory and hypothesis-generating, given the reduced sample size after stratification and the number of predictors included. The exploratory subgroup analyses within the VFMI population provided additional insights into the functional heterogeneity of VFMI subtypes. Within the laterality subgroup, laryngeal vestibule secretion accumulation was the sole indicator independently associated with bilateral (vs. unilateral) VFMI (OR = 2.454, *p* = 0.006), suggesting that symmetric bilateral neural involvement produces more pervasive supraglottic secretion retention than unilateral impairment alone. Within the severity subgroup, pyriform sinus residue rating was the sole indicator independently associated with complete vocal fold immobility (vs. hypomotility) (OR = 2.093, *p* = 0.038), consistent with a gradient relationship between the extent of motor output disruption and pharyngeal propulsive failure. These subtype-specific associations—distinct from those identified in the primary model—suggest that VFMI should not be treated as a uniform entity. It is essential to emphasize at the outset that both subgroup analyses were conducted within the VFMI stratum (*n* = 220) using 16-predictor models with limited post-stratification statistical power; accordingly, all findings should be treated as strictly hypothesis-generating and interpreted with caution. With this caveat as the primary interpretive frame, the observed laterality and severity associations may tentatively suggest that different VFMI subtypes are accompanied by distinct swallowing functional profiles. Should these patterns be replicated in larger, subgroup-powered cohorts, they could potentially provide preliminary directions for considering subtype-informed differences in rehabilitation assessment—though they do not currently constitute sufficient evidence to guide rehabilitation target selection in clinical practice.

### 4.2 Comparison with previous studies

Previous studies have observed the coexistence of dysphagia and VFMI in patients with stroke and other neurological diseases, but most have focused on single functional indicators or specific clinical outcomes, such as aspiration or pneumonia incidence (27, 28). In comparison, the advantage of this study lies in conducting multidimensional, structured analysis of swallowing function based on a unified FEES assessment framework, thereby enabling a more comprehensive depiction of the association patterns between swallowing dysfunction and vocal cord movement disorders. The study results to some extent expand upon previous findings, emphasizing the important role of pharyngeal phase motor function and secretion management capacity in the functional phenotype of VFMI.

It should be noted that not all swallowing function indicators maintained statistical significance in multivariable analysis, a result suggesting that the functional manifestations of VFMI exhibit certain selectivity and heterogeneity, further supporting the view that it is not simply equivalent to “severe dysphagia.”

### 4.3 Clinical significance and practice implications

The clinical significance of this study lies not in proposing new screening or prediction tools, but in providing a functionally integrated perspective for clinical practice. In patients with brain injury, VFMI is often regarded as a functional abnormality confined to the larynx, with primary focus on its impact on phonation or airway closure (29, 30). However, this study suggests that VFMI commonly coexists with pharyngeal phase motor dysfunction and reduced secretion management capacity, and should be understood as an important component within the broader swallowing and airway-protective dysfunction spectrum arising from shared brainstem network impairment.

The practical clinical value of the present findings lies specifically in scenarios where vocal fold status is not simultaneously available—the precise settings in which restricted pharyngeal wall movement and pyriform sinus secretion pooling can function as meaningful clinical clues prompting further evaluation of possible concurrent VFMI. First, non-FEES clinical swallowing screening: In bedside swallowing screening settings (e.g., water swallow tests or standardized swallowing assessment protocols), the examining clinician typically does not have real-time endoscopic access to vocal fold status. When a prior FEES report documents restricted pharyngeal wall movement or pyriform sinus secretion pooling, our findings suggest that VFMI should be proactively incorporated as a concurrent functional consideration—informing decisions regarding the urgency and scope of formal laryngoscopic re-evaluation—even in the absence of overt dysphonia at the time of screening. Second, transferred patients lacking video-documented vocal fold findings: Patients transferred from other institutions frequently arrive with textual FEES reports describing pharyngeal findings, but without archived video documentation of vocal fold status. In such cases, clinicians at the receiving center cannot directly verify vocal fold mobility from available records. Documented restricted pharyngeal wall movement or pyriform sinus secretion pooling in transfer records should therefore be regarded as a clinical clue to likely concurrent VFMI, warranting prioritized repeat FEES with explicit vocal fold examination upon admission. Third, FEES indication in patients without dysphonia: Brain injury patients without prominent voice disturbance are often not flagged under conventional clinical pathways for systematic vocal fold evaluation. Yet the present data demonstrate that VFMI frequently co-manifests with restricted pharyngeal wall movement and pyriform sinus secretion pooling even in the absence of overt phonatory symptoms, as a consequence of shared neural substrate impairment. When FEES reveals these pharyngeal findings in a patient without dysphonia, the vocal fold examination component should be explicitly prioritized, documented, and interpreted—rather than treated as incidental—given the elevated likelihood of concurrent VFMI. Fourth, stroboscopy or laryngoscopy without parallel swallowing assessment: In otolaryngology or voice clinic settings where laryngoscopy or stroboscopy is performed for voice-related indications without concurrent swallowing evaluation, the bidirectional nature of pharyngolaryngeal co-manifestation identified in this study supports proactive referral for FEES-based swallowing assessment when VFMI is detected in brain injury patients, even when swallowing complaints are not the primary presenting concern.

Across all four scenarios, restricted pharyngeal wall movement and pyriform sinus secretion pooling serve as associated clinical clues—not as standalone VFMI screening criteria—that support a more functionally integrated interpretation of pharyngolaryngeal status. This integrated perspective may help: (1) reduce the rate of missed VFMI diagnosis, particularly in patients without obvious voice disturbances; (2) provide a more comprehensive functional basis for decisions regarding tracheostomy decannulation timing; and (3) inform the design of rehabilitation programs that incorporate cough rehabilitation and airway-protective training alongside swallowing intervention, rather than treating VFMI as an isolated laryngeal problem.

It is necessary to address explicitly how participant characteristics—particularly consciousness level and FEES timing—may have influenced our findings. Regarding FEES timing: all assessments were conducted within 48–72 h of admission to our specialized neurological rehabilitation center, which primarily receives patients in the subacute-to-chronic recovery phase (median disease duration: 77.0–89.5 days). This timing reflects institutional admission patterns rather than acute post-onset assessment, and should be considered when generalizing these findings to acute-phase populations. Regarding consciousness level: patients in coma who could not safely undergo FEES were excluded during screening. For minimally conscious patients, FEES was adapted to resting-state endoscopic observation of vocal fold position and secretion status; formal bolus challenges were administered only when protective reflexes were sufficiently intact to ensure safety. This protocol adaptation means that swallowing indicators derived from bolus challenges may be underrepresented or missing in the minimally conscious subgroup, which constitutes a potential source of differential data completeness. Importantly, consciousness level did not differ significantly between the VFMI and non-VFMI groups at baseline (*p* > 0.05, Table 1), and did not emerge as an independent predictor in the multivariable model, suggesting that the functional coupling between restricted pharyngeal wall movement, pyriform sinus secretion pooling, and VFMI reflects a shared neural substrate injury not simply mediated by arousal level. Furthermore, the exploratory subgroup analyses (Section 3.4) found no significant association between consciousness level and VFMI severity subtype (hypomotility vs. complete immobility) or laterality (unilateral vs. bilateral), suggesting that the swallowing-VFMI co-manifestation patterns identified in this study are relatively robust across the consciousness spectrum represented in our cohort. Nevertheless, given the absence of standardized GCS or NIHSS scores and the coarse three-tier consciousness classification used, residual confounding from neurological severity cannot be fully excluded, as detailed in Section 4.4. This is particularly relevant given that the majority of patients in our cohort were in the subacute-to-chronic recovery phase (median disease duration: 77.0–89.5 days), a period during which consciousness level and swallowing-laryngeal function may follow distinct and partially independent recovery trajectories. Nevertheless, clinicians should remain attentive to the need for adapting FEES protocols to the patient’s consciousness level: in minimally conscious patients, resting-state endoscopic observation of vocal fold mobility and secretion accumulation may still provide clinically meaningful information regarding the presence of VFMI and associated airway protection risks, even when formal bolus challenges cannot be safely administered. This adaptation of the clinical pathway to consciousness level represents an important practical consideration that should be integrated into bedside assessment protocols for patients with brain injury across the continuum of neurological recovery.

### 4.4 Methodological strengths and study limitations

This study has certain limitations. First, the cross-sectional design can only reveal associations and cannot determine causal direction; therefore, clinical pathway recommendations still require validation through prospective studies. Second, the single-center design limits the generalizability of the results. Study subjects were recruited from a specialized neurological hospital, where disease severity may be higher than that in general hospitals or community medical institutions. The VFMI detection rate in this study was 78.6%, which is higher than that reported in previous literature (22, 31). This may be related to the following factors: (1) more severely ill referred patients treated at specialized hospitals; and (2) systematic fiberoptic laryngoscopy, which can identify vocal fold movement restriction with subtle symptoms. Therefore, the strength of association between restricted pharyngeal wall movement (OR = 2.158) and pyriform sinus secretion pooling (PS_MAX: OR = 2.165) with VFMI may not be directly extrapolatable to mild patient populations. Additionally, the single-center design cannot exclude the influence of center-specific factors, such as assessment practices. Third, detailed endotracheal intubation history, including duration of intubation, mechanical ventilation history, and time since extubation, was not consistently available. Because prior airway instrumentation may contribute to vocal fold hypomobility or fixation through laryngeal injury, arytenoid dislocation, cricoarytenoid joint fixation, or post-extubation laryngeal dysfunction, residual confounding related to intubation history cannot be excluded. Fourth, Standardized neurological severity scores such as the Glasgow Coma Scale (GCS) or National Institutes of Health Stroke Scale (NIHSS) were not systematically recorded at the time of FEES assessment. Although a three-tier categorical classification of consciousness level (alert, minimally conscious, coma) was included as an available proxy for neurological status and adjusted for in the multivariable model, this coarse classification cannot fully capture the severity and heterogeneity of brain injury across the subacute-to-chronic recovery continuum represented in this cohort. Specifically, within-category variability in neurological severity—particularly within the minimally conscious tier, which encompasses a wide spectrum of residual responsiveness—may introduce residual confounding that our model cannot fully account for. Future prospective studies should incorporate standardized GCS or NIHSS scores recorded contemporaneously with FEES assessment, and consider stratified analyses by both consciousness level and disease chronicity, to enable more precise evaluation of whether and how these factors independently modulate the severity of VFMI or dysphagia. Future prospective, multicenter studies should incorporate detailed airway intervention history and standardized neurological severity scales to improve confounding control and further validate the generalizability and causal relevance of the observed associations.

However, the following methodological strengths partially mitigate the above limitations: (1) Consecutive enrollment design: This study included all eligible patients during the period from June 2024 to May 2025 (316 screened, 280 included), avoiding selection bias and authentically reflecting the patient distribution characteristics of this center; (2) Adequate sample size: 280 patients provided sufficient statistical power for multivariable analysis, improving the stability of effect estimation; (3) Standardized assessment: Uniform equipment and procedures were employed with blinded interpretation by dual evaluators, ensuring measurement consistency; (4) Adequate confounding adjustment: The multivariable model included confounding factors such as age, sex, diagnosis type, lesion location, disease duration, level of consciousness, and tracheostomy status;(5) Composite variable construction: Bilateral pyriform sinus secretion pooling variables were combined into a composite maximum-score variable (PS_MAX) to avoid arbitrary single-side exclusion and retain bilateral clinical information.

The findings of this study still require validation in multicenter populations across different healthcare levels and geographical regions to confirm the universality of associations. Additionally, the diagnosis of VFMI in this study was based solely on fiberoptic endoscopic observation of vocal fold position and mobility, without laryngeal electromyography (L-EMG) confirmation. Consequently, we cannot exclude the possibility that some cases of apparent hypomotility reflect mechanical causes—such as arytenoid dislocation or cricoarytenoid joint fixation—rather than true neurogenic impairment, and conversely, that some cases of complete fixation may represent non-neurogenic mechanical restriction. The current classification therefore represents a descriptive, endoscopy-based phenotyping of vocal fold motor function rather than an etiologically confirmed diagnosis of neurogenic paralysis. Future studies should incorporate L-EMG to enable etiological differentiation. Additionally, some subjective indicator assessments may benefit from complementary objective measures, such as dynamic MRI, in future investigations.

## 5 Conclusion

In summary, this study demonstrates that in patients with brain injury, restricted pharyngeal wall movement and pyriform sinus secretion pooling co-manifest with VFMI as interconnected expressions of shared neural substrate injury. This finding reinforces the understanding that VFMI should not be viewed as an isolated laryngeal functional abnormality, but as a co-manifestation within the broader swallowing and airway-protective dysfunction spectrum—integrating both pharyngeal motor dysfunction and impaired cough-related airway protection—arising from common brainstem network impairment. The clinical translational value of this study lies in identifying concrete scenarios where FEES-derived pharyngeal findings can serve as actionable clinical clues to concurrent VFMI—specifically in settings where vocal fold status is not simultaneously available, such as non-FEES bedside swallowing screening, assessment of transferred patients lacking video-documented vocal fold findings, evaluation of brain injury patients without overt dysphonia, and laryngoscopy performed without parallel swallowing assessment. In these contexts, restricted pharyngeal wall movement and pyriform sinus secretion pooling should be interpreted not as standalone screening criteria, but as integrated clinical signals that prompt more systematic evaluation of pharyngolaryngeal function and support comprehensive, coordinated rehabilitation planning that addresses swallowing dysfunction, cough impairment, and broader airway-protective deficits arising from brain injury.

## Data Availability

The original contributions presented in the study are included in the article/Supplementary material, further inquiries can be directed to the corresponding author.

## References

[1] Feigin VL Vos T Nichols E Owolabi MO Carroll WM Dichgans M . The global burden of neurological disorders: translating evidence into policy. Lancet Neurol. (2020) 19:255–65. doi: 10.1016/S1474-4422(19)30411-9, 31813850 PMC9945815

[2] Schefold JC Berger D Zürcher P Lensch M Perren A Jakob SM . Dysphagia in mechanically ventilated ICU patients (DYnAMICS): a prospective observational trial. Crit Care Med. (2017) 45:2061–9. doi: 10.1097/CCM.0000000000002765 29023260

[3] Francis DO Williamson K Hovis K Gelbard A Merati AL Penson DF . Effect of injection augmentation on need for framework surgery in unilateral vocal fold paralysis. Laryngoscope. (2016) 126:128–34. doi: 10.1002/lary.25431, 26153268 PMC4704988

[4] Molfenter SM Steele CM. Physiological variability in the deglutition literature: hyoid and laryngeal kinematics. Dysphagia. (2011) 26:67–74. doi: 10.1007/s00455-010-9309-x, 20927634 PMC3756522

[5] Steele CM Miller AJ. Sensory input pathways and mechanisms in swallowing: a review. Dysphagia. (2010) 25:323–33. doi: 10.1007/s00455-010-9301-5, 20814803 PMC2992653

[6] Arnold M Liesirova K Broeg-Morvay A Meisterernst J Schlager M Mono ML . Dysphagia in acute stroke: incidence, burden and impact on clinical outcome. PLoS One. (2016) 11:e0148424. doi: 10.1371/journal.pone.0148424, 26863627 PMC4749248

[7] Langmore SE. History of fiberoptic endoscopic evaluation of swallowing for evaluation and management of pharyngeal dysphagia: changes over the years. Dysphagia. (2017) 32:27–38. doi: 10.1007/s00455-016-9775-x 28101663

[8] Jean A. Brain stem control of swallowing: neuronal network and cellular mechanisms. Physiol Rev. (2001) 81:929–69. doi: 10.1152/physrev.2001.81.2.929 11274347

[9] Kim H Chung CS Lee KH Robbins J. Aspiration subsequent to a pure medullary infarction: lesion sites, clinical variables, and outcome. Arch Neurol. (2000) 57:478–83. doi: 10.1001/archneur.57.4.478, 10768620

[10] Bhattacharyya N Kotz T Shapiro J. Dysphagia and aspiration with unilateral vocal cord immobility: incidence, characterization, and response to surgical treatment. Ann Otol Rhinol Laryngol. (2002) 111:672–9. doi: 10.1177/000348940211100803, 12184586

[11] Heitmiller RF Tseng E Jones B. Prevalence of aspiration and laryngeal penetration in patients with unilateral vocal fold motion impairment. Dysphagia. (2000) 15:184–7. doi: 10.1007/s004550000026, 11014880

[12] Kelchner LN Brehm SB Weinrich B Middendorf J deAlarcon A Levin L . Perceptual evaluation of severe pediatric voice disorders: rater reliability using the consensus auditory perceptual evaluation of voice. J Voice. (2010) 24:441–9. doi: 10.1016/j.jvoice.2008.09.004, 19135856

[13] Cates DJ Venkatesan NN Strong B Kuhn MA Belafsky PC. Effect of vocal fold medialization on dysphagia in patients with unilateral vocal fold immobility. Otolaryngol Head Neck Surg. (2016) 155:454–7. doi: 10.1177/0194599816645765, 27165683

[14] Steele CM Grace-Martin K. Reflections on clinical and statistical use of the penetration-aspiration scale. Dysphagia. (2017) 32:601–16. doi: 10.1007/s00455-017-9809-z 28534064 PMC5608795

[15] Swan K Speyer R Heijnen BJ Wagg B Cordier R. Living with oropharyngeal dysphagia: effects of bolus modification on health-related quality of life—a systematic review. Qual Life Res. (2015) 24:2447–56. doi: 10.1007/s11136-015-0990-y 25869989

[16] Speyer R Baijens L Heijnen M Zwijnenberg I. Effects of therapy in oropharyngeal dysphagia by speech and language therapists: a systematic review. Dysphagia. (2010) 25:40–65. doi: 10.1007/s00455-009-9239-7, 19760458 PMC2846331

[17] Cichero JA Lam P Steele CM Hanson B Chen J Dantas RO . Development of international terminology and definitions for texture-modified foods and thickened fluids used in dysphagia management: the IDDSI framework. Dysphagia. (2017) 32:293–314. doi: 10.1007/s00455-016-9758-y, 27913916 PMC5380696

[18] Wirth R Dziewas R Beck AM Clavé P Hamdy S Heppner HJ . Oropharyngeal dysphagia in older persons–from pathophysiology to adequate intervention: a review and summary of an international expert meeting. Clin Interv Aging. (2016) 11:189–208. doi: 10.2147/CIA.S97481, 26966356 PMC4770066

[19] Francis DO McPheeters ML Noud M Penson DF Feurer ID. Voice-related patient-reported outcome measures: a systematic review of instrument development and validation. J Speech Lang Hear Res. (2016) 59:62–75. doi: 10.1044/2015_JSLHR-S-15-0113 28030869 PMC5533561

[20] Rosen CA Gartner-Schmidt J Hathaway B Simpson CB Postma GN Courey M . A nomenclature paradigm for benign midmembranous vocal fold lesions. Laryngoscope. (2012) 122:1335–41. doi: 10.1002/lary.22421, 22522621

[21] Macht M Wimbish T Clark BJ Benson AB Burnham EL Williams A . Postextubation dysphagia is persistent and associated with poor outcomes in survivors of critical illness. Crit Care. (2011) 15:R231. doi: 10.1186/cc10472, 21958475 PMC3334778

[22] González-Fernández M Ottenstein L Atanelov L Christian AB. Dysphagia after stroke: an overview. Curr Phys Med Rehabil Rep. (2013) 1:187–96. doi: 10.1007/s40141-013-0017-y, 24977109 PMC4066736

[23] Rubin AD Sataloff RT. Vocal fold paresis and paralysis: what the thyroid surgeon should know. Surg Oncol Clin N Am. (2008) 17:175–96. doi: 10.1016/j.soc.2007.10.007, 18177806

[24] Patel RR Awan SN Barkmeier-Kraemer J Courey M Deliyski D Eadie T . Recommended protocols for instrumental assessment of voice: American speech-language-hearing association expert panel to develop a protocol for instrumental assessment of vocal function. Am J Speech Lang Pathol. (2018) 27:887–905. doi: 10.1044/2018_AJSLP-17-0009, 29955816

[25] Steele CM Peladeau-Pigeon M Barrett E Wolkin TS. The risk of penetration-aspiration related to residue in the pharynx. Am J Speech Lang Pathol. (2020) 29:1608–17. doi: 10.1044/2020_AJSLP-20-00042, 32598168 PMC7893525

[26] Phua SY McGarvey LP Ngu MC Ing AJ. Patients with gastro-oesophageal reflux disease and cough have impaired laryngopharyngeal mechanosensitivity. Thorax. (2005) 60:488–91. doi: 10.1136/thx.2004.033894, 15923249 PMC1747421

[27] Macht M Wimbish T Bodine C Moss M. ICU-acquired swallowing disorders. Crit Care Med. (2013) 41:2396–405. doi: 10.1097/CCM.0b013e31829caf33, 23939361

[28] Cabib C Ortega O Kumru H Palomeras E Vilardell N Alvarez-Berdugo D . Neurorehabilitation strategies for poststroke oropharyngeal dysphagia: from compensation to the recovery of swallowing function. Ann N Y Acad Sci. (2016) 1380:121–38. doi: 10.1111/nyas.13135, 27398981

[29] Hadjikoutis S Eccles R Wiles CM. Coughing and choking in motor neuron disease. J Neurol Neurosurg Psychiatry. (2000) 68:601–4. doi: 10.1136/jnnp.68.5.601, 10766890 PMC1736915

[30] Sulica L. The natural history of idiopathic unilateral vocal fold paralysis: evidence and problems. Laryngoscope. (2008) 118:1303–7. doi: 10.1097/MLG.0b013e31816f27ee, 18496160

[31] Suntrup-Krueger S Kemmling A Warnecke T Hamacher C Oelenberg S Niederstadt T . The impact of lesion location on dysphagia incidence, pattern and complications in acute stroke. Part 2: oropharyngeal residue, swallow and cough response, and pneumonia. Eur J Neurol. (2017) 24:867–74. doi: 10.1111/ene.13307, 28449405

