# Supplementary material for: Co-manifestation of swallowing dysfunction indicators and vocal fold motion impairment within a shared neural substrate in patients with brain injury: a cross-sectional study
Source: Front Neurol. 2026 Jun 24;17:1790412. doi: 10.3389/fneur.2026.1790412 (PMC13341431; doi:10.3389/fneur.2026.1790412)
Supplement: Supplementary file 1 [file Table_1.DOCX]

Supplementary Material

**
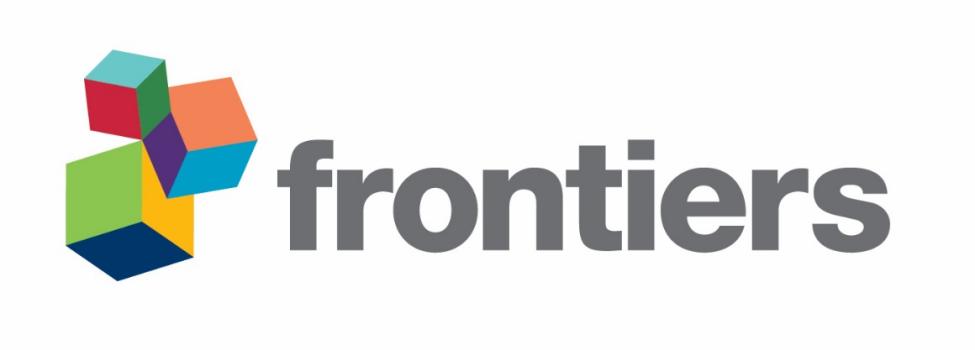
**

**Supplementary Table 1.** Univariate Analysis of Swallowing Function Indicators

| **Normal Vocal Cord Group (n=60)** | **Abnormal Vocal Cord Group (n=220)** |  |  |  |  |  |  |  |
| --- | --- | --- | --- | --- | --- | --- | --- | --- |
| Nasopharyngeal Secretions | Normal | 14 (23.33333%) | 17 (7.72727%) | p=0.0006041 (Mann-Whitney U test) | Effect Size (Cliff's Delta): -0.1986364 Effect Size (rank biserial correlation): -0.199 | 95% CI: [-0.31510335, -0.07627527] |  | Wilcoxon rank-sum statistic (W): 5289 |
|  | Trace | 44 (73.33333%) | 182 (82.72727%) |  |  |  |  |  |
|  | Mild | 2 (3.33333%) | 20 (9.09091%) |  |  |  |  |  |
|  | Moderate | 0 (0.00000%) | 1 (0.45455%) |  |  |  |  |  |
|  | Severe | 0 (0.00000%) | 0 (0.00000%) |  |  |  |  |  |
| Vallecular Secretions | Normal | 22 (36.66667%) | 15 (6.81818%) | p=5.852e-08 (Mann-Whitney U test) | Effect Size (Cliff's Delta): -0.4332576 Effect Size (rank biserial correlation): 0.433 | 95% CI: [-0.5660881, -0.2784980] |  | Wilcoxon rank-sum statistic (W): 3740.5 |
|  | Trace | 26 (43.33333%) | 95 (43.18182%) |  |  |  |  |  |
|  | Mild | 8 (13.33333%) | 63 (28.63636%) |  |  |  |  |  |
|  | Moderate | 1 (1.66667%) | 28 (12.72727%) |  |  |  |  |  |
|  | Severe | 3 (5.00000%) | 19 (8.63636%) |  |  |  |  |  |
| Pyriform Sinus Secretions L | Normal | 18 (30.00000%) | 15 (6.81818%) | p=3.968e-09 (Mann-Whitney U test) | Effect Size (Cliff's Delta): -0.4799242 Effect Size (rank biserial correlation): 0.48 | 95% CI: [-0.6107455, -0.3235992] |  | Wilcoxon rank-sum statistic (W): 3432.5 |
|  | Trace | 27 (45.00000%) | 53 (24.09091%) |  |  |  |  |  |
|  | Mild | 8 (13.33333%) | 79 (35.90909%) |  |  |  |  |  |
|  | Moderate | 4 (6.66667%) | 51 (23.18182%) |  |  |  |  |  |
|  | Severe | 3 (5.00000%) | 22 (10.00000%) |  |  |  |  |  |
| Pyriform Sinus Secretions R | Normal | 18 (30.00000%) | 14 (6.36364%) | p=2.066e-09 (Mann-Whitney U test) | Effect Size (Cliff's Delta): -0.4882576 Effect Size (rank biserial correlation): 0.488 | 95% CI: [-0.6187236, -0.3315829] |  | Wilcoxon rank-sum statistic (W): 3377.5 |
|  | Trace | 27 (45.00000%) | 52 (23.63636%) |  |  |  |  |  |
|  | Mild | 8 (13.33333%) | 82 (37.27273%) |  |  |  |  |  |
|  | Moderate | 4 (6.66667%) | 49 (22.27273%) |  |  |  |  |  |
|  | Severe | 3 (5.00000%) | 23 (10.45455%) |  |  |  |  |  |
| Laryngeal Vestibule Secretions | Normal | 49 (81.66667%) | 112 (50.90909%) | p=2.996e-05 (Mann-Whitney U test) | Effect Size (Cliff's Delta): -0.3137879 Effect Size (rank biserial correlation): 0.314 | 95% CI: [-0.4289591, -0.1885783] |  | Wilcoxon rank-sum statistic (W): 4529 |
|  | Trace | 7 (11.66667%) | 58 (26.36364%) |  |  |  |  |  |
|  | Mild | 2 (3.33333%) | 26 (11.81818%) |  |  |  |  |  |
|  | Moderate | 0 (0.00000%) | 16 (7.27273%) |  |  |  |  |  |
|  | Severe | 2 (3.33333%) | 8 (3.63636%) |  |  |  |  |  |
| Murray Grading | Grade 0 | 48 (80.00000%) | 101 (45.90909%) | p=8.515e-06 (Mann-Whitney U test) | Effect Size (Cliff's Delta): -0.342803 Effect Size (rank biserial correlation): 0.343 | 95% CI: [-0.4617206, -0.2117807] |  | Wilcoxon rank-sum statistic (W): 4337.5 |
|  | Grade 1 | 5 (8.33333%) | 50 (22.72727%) |  |  |  |  |  |
|  | Grade 2 | 5 (8.33333%) | 45 (20.45455%) |  |  |  |  |  |
|  | Grade 3 | 2 (3.33333%) | 24 (10.90909%) |  |  |  |  |  |
| Swallowing Frequency | ≥3 times/min:1 | 40 (66.66667%) | 88 (40.00000%) | p=0.0003305 (Mann-Whitney U test) | Effect Size (Cliff's Delta): 0.2730303 Effect Size (rank biserial correlation): 0.273 | 95% CI: [-0.4058718, -0.1288908] |  | Wilcoxon rank-sum statistic (W): 4798 |
|  | <3 times/min:2 | 17 (28.33333%) | 108 (49.09091%) |  |  |  |  |  |
|  | No voluntary swallowing:3 | 3 (5.00000%) | 24 (10.90909%) |  |  |  |  |  |
| Velopharyngeal Closure | Normal 1 | 12 (20.00000%) | 13 (5.90909%) | p=0.001308 (Mann-Whitney U test) | Effect Size (Cliff's Delta): -0.1713636 Effect Size (rank biserial correlation): 0.171 | 95% CI: [-0.28413993, -0.05391025] |  | Wilcoxon rank-sum statistic (W): 5469 |
|  | Incomplete 2 | 46 (76.66667%) | 190 (86.36364%) |  |  |  |  |  |
|  | Cannot 3 | 2 (3.33333%) | 17 (7.72727%) |  |  |  |  |  |
| Tongue Base Retraction | Normal 1 | 8 (13.33333%) | 9 (4.09091%) | p=0.003866 (Mann-Whitney U test) | Effect Size (Cliff's Delta): -0.1510606 Effect Size (rank biserial correlation): 0.151 | 95% CI: [-0.25162308, -0.04727115] |  | Wilcoxon rank-sum statistic (W): 5603 |
|  | Reduced 2 | 50 (83.33333%) | 188 (85.45455%) |  |  |  |  |  |
|  | Absent 3 | 2 (3.33333%) | 23 (10.45455%) |  |  |  |  |  |
| Pharyngeal Wall Movement | Normal 1 | 13 (21.66667%) | 0 (0.00000%) | p=0.0001754 (Mann-Whitney U test) | Effect Size (Cliff's Delta): -0.279697 Effect Size (rank biserial correlation): 0.28 | 95% CI: [-0.4280450, -0.1166721] |  | Wilcoxon rank-sum statistic (W): 4754 |
|  | Reduced 2 | 26 (43.33333%) | 104 (47.27273%) |  |  |  |  |  |
|  | Absent 3 | 21 (35.00000%) | 116 (52.72727%) |  |  |  |  |  |
| Gag Reflex (Sensation, light touch to pharyngeal wall) | Normal 1 | 21 (35.00000%) | 34 (15.45455%) | p=0.000304 (Mann-Whitney U test) | Effect Size (Cliff's Delta): -0.2345455 Effect Size (rank biserial correlation): 0.235 | 95% CI: [-0.3598301, -0.1009526] |  | Wilcoxon rank-sum statistic (W): 5052 |
|  | Weak response 2 | 38 (63.33333%) | 168 (76.36364%) |  |  |  |  |  |
|  | No response 3 | 1 (1.66667%) | 18 (8.18182%) |  |  |  |  |  |
| Cough Reflex | Normal 1 | 25 (41.66667%) | 51 (23.18182%) | p=0.004598 (Mann-Whitney U test) | Effect Size (Cliff's Delta): -0.1906061 Effect Size (rank biserial correlation): 0.191 | 95% CI: [-0.32564258, -0.04794905] |  | Wilcoxon rank-sum statistic (W): 5342 |
|  | Delayed 2 | 34 (56.66667%) | 162 (73.63636%) |  |  |  |  |  |
|  | Absent 3 | 1 (1.66667%) | 7 (3.18182%) |  |  |  |  |  |
| Cough Effectiveness | Complete 1 | 3 (5.00000%) | 0 (0.00000%) | p=9.977e-07 (Mann-Whitney U test) | Effect Size (Cliff's Delta): -0.3543182 Effect Size (rank biserial correlation): 0.354 | 95% CI: [-0.4834446, -0.2101008] |  | Wilcoxon rank-sum statistic (W): 4261.5 |
|  | Some 2 | 24 (40.00000%) | 35 (15.90909%) |  |  |  |  |  |
|  | Slight 3 | 30 (50.00000%) | 146 (66.36364%) |  |  |  |  |  |
|  | Ineffective 4 | 3 (5.00000%) | 39 (17.72727%) |  |  |  |  |  |
| Vallecular Residue Rating | None 0 | 12 (20.00000%) | 9 (4.09091%) | p=4.779e-05 (Mann-Whitney U test) | Effect Size (Cliff's Delta): -0.3296212 Effect Size (rank biserial correlation): 0.33 | 95% CI: [-0.4688156, -0.1744525] |  | Wilcoxon rank-sum statistic (W): 4424.5 |
|  | Trace 1 | 21 (35.00000%) | 64 (29.09091%) |  |  |  |  |  |
|  | Mild 2 | 20 (33.33333%) | 76 (34.54545%) |  |  |  |  |  |
|  | Moderate 3 | 4 (6.66667%) | 40 (18.18182%) |  |  |  |  |  |
|  | Severe 4 | 3 (5.00000%) | 31 (14.09091%) |  |  |  |  |  |
| Piriform Sinus Residue Rating | None 0 | 7 (11.66667%) | 5 (2.27273%) | p=1.373e-05 (Mann-Whitney U test) | Effect Size (Cliff's Delta): -0.3544697 Effect Size (rank biserial correlation): 0.354 | 95% CI: [-0.4961119, -0.1944498] |  | Wilcoxon rank-sum statistic (W): 4260.5 |
|  | Trace 1 | 28 (46.66667%) | 57 (25.90909%) |  |  |  |  |  |
|  | Mild 2 | 13 (21.66667%) | 64 (29.09091%) |  |  |  |  |  |
|  | Moderate 3 | 7 (11.66667%) | 56 (25.45455%) |  |  |  |  |  |
|  | Severe 4 | 5 (8.33333%) | 38 (17.27273%) |  |  |  |  |  |
| Rosenbek (Penetration-Aspiration Scale) | Grade 1 | 24 (40.00000%) | 23 (10.45455%) | p=6.566e-08 (Mann-Whitney U test) | Effect Size (Cliff's Delta): -0.4487121 Effect Size (rank biserial correlation): 0.449 | 95% CI: [-0.5817023, -0.2923567] |  | Wilcoxon rank-sum statistic (W): 3638.5 |
|  | Grade 2 | 8 (13.33333%) | 28 (12.72727%) |  |  |  |  |  |
|  | Grade 3 | 1 (1.66667%) | 2 (0.90909%) |  |  |  |  |  |
|  | Grade 4 | 17 (28.33333%) | 53 (24.09091%) |  |  |  |  |  |
|  | Grade 5 | 3 (5.00000%) | 30 (13.63636%) |  |  |  |  |  |
|  | Grade 6 | 1 (1.66667%) | 24 (10.90909%) |  |  |  |  |  |
|  | Grade 7 | 3 (5.00000%) | 34 (15.45455%) |  |  |  |  |  |
|  | Grade 8 | 3 (5.00000%) | 26 (11.81818%) |  |  |  |  |  |
| Nutrition | Tube feeding | 39 (65.00000%) | 181 (82.27273%) | p=0.0066716 (Chi-square test) | Effect Size (Phi coefficient): 0.1621212 | Relative Risk (RR): 0.7900552 | Odds Ratio (OR): 0.4001579 | Chi-square statistic (χ²): 7.3593205 |
|  | Oral intake | 21 (35.00000%) | 39 (17.72727%) |  |  |  |  |  |
| Swelling | Yes | 4 (6.666667%) | 47 (21.363636%) | p=0.0152731 (Chi-square test) | Effect Size (Phi coefficient): 0.1449715 | Relative Risk (RR): 0.3120567 | Odds Ratio (OR): 0.2629179 | Chi-square statistic (χ²): 5.8846881 |
|  | No | 56 (93.333333%) | 173 (78.636364%) |  |  |  |  |  |

**Supplementary Table2.** Spearman Correlation between Pyriform Sinus Secretions Retention (Left) and Other Indicators

| Correlated Variables | Spearman Correlation Coefficient (r) | Correlation Intensity Classification |
| --- | --- | --- |
| Pyriform Sinus Secretions Retention (Right) | 0.974 | Extremely High Correlation |
| Vallecular Secretions Retention | 0.866 | High Correlation |
| Murray Grade Score | 0.797 | High Correlation |
| Pyriform Sinus Residue Rating | 0.754 | High Correlation |
| Rosenbek Penetration-Aspiration Scale Score | 0.718 | High Correlation |
| Laryngeal Vestibule Secretions Retention | 0.705 | High Correlation |
| Vallecular Residue Rating | 0.668 | Moderate Correlation |
| Cough Effectiveness | 0.577 | Moderate Correlation |
| Nasopharyngeal Secretions Retention | 0.503 | Moderate Correlation |

**Supplementary Figure1.**


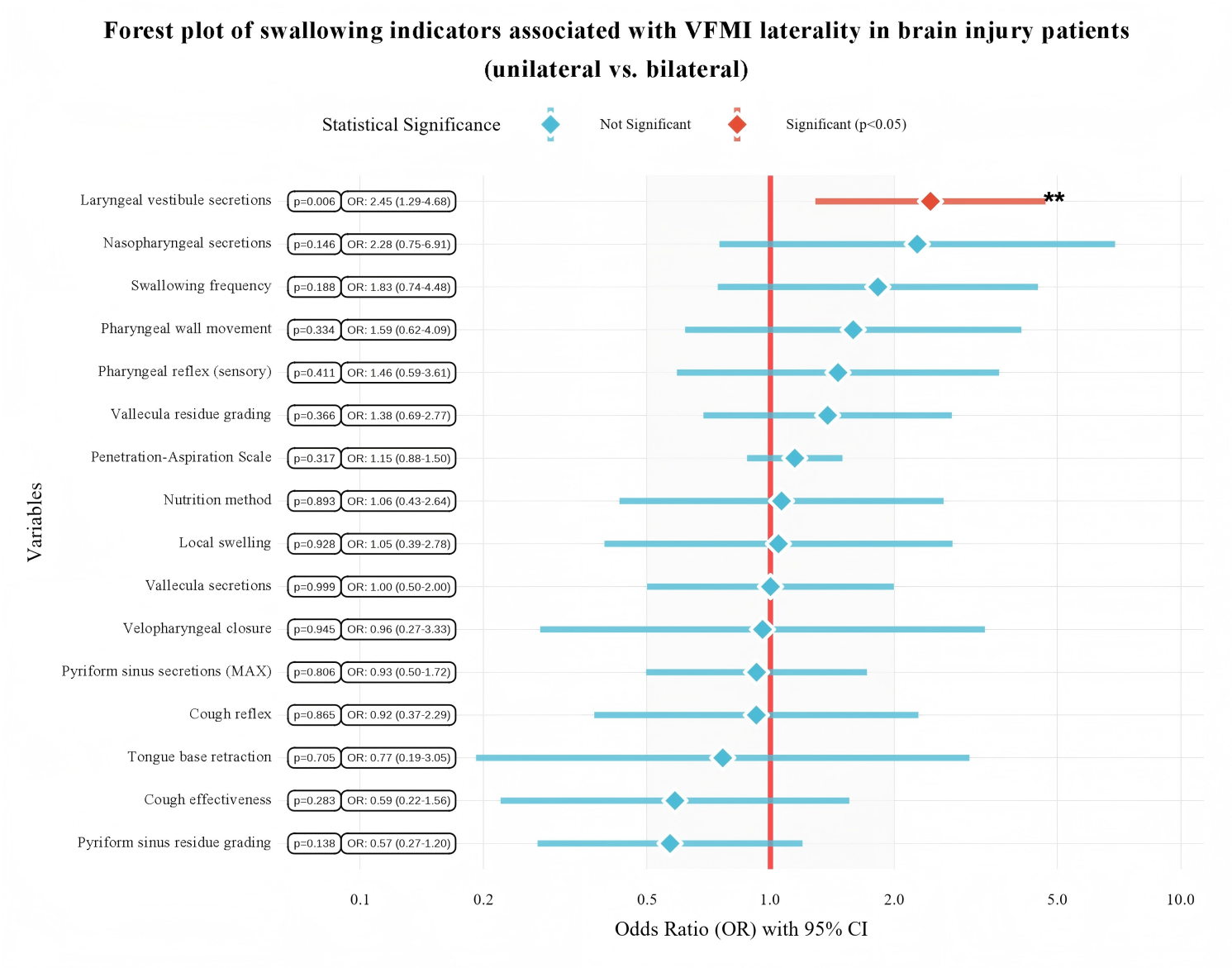


**Supplementary Figure 1.** Red diamonds indicate statistically significant variables (P < 0.05); blue diamonds indicate non-significant variables. The vertical red line represents OR = 1.0. Analysis restricted to patients with confirmed VFMI (n = 220).

**Supplementary Figure2.**

**
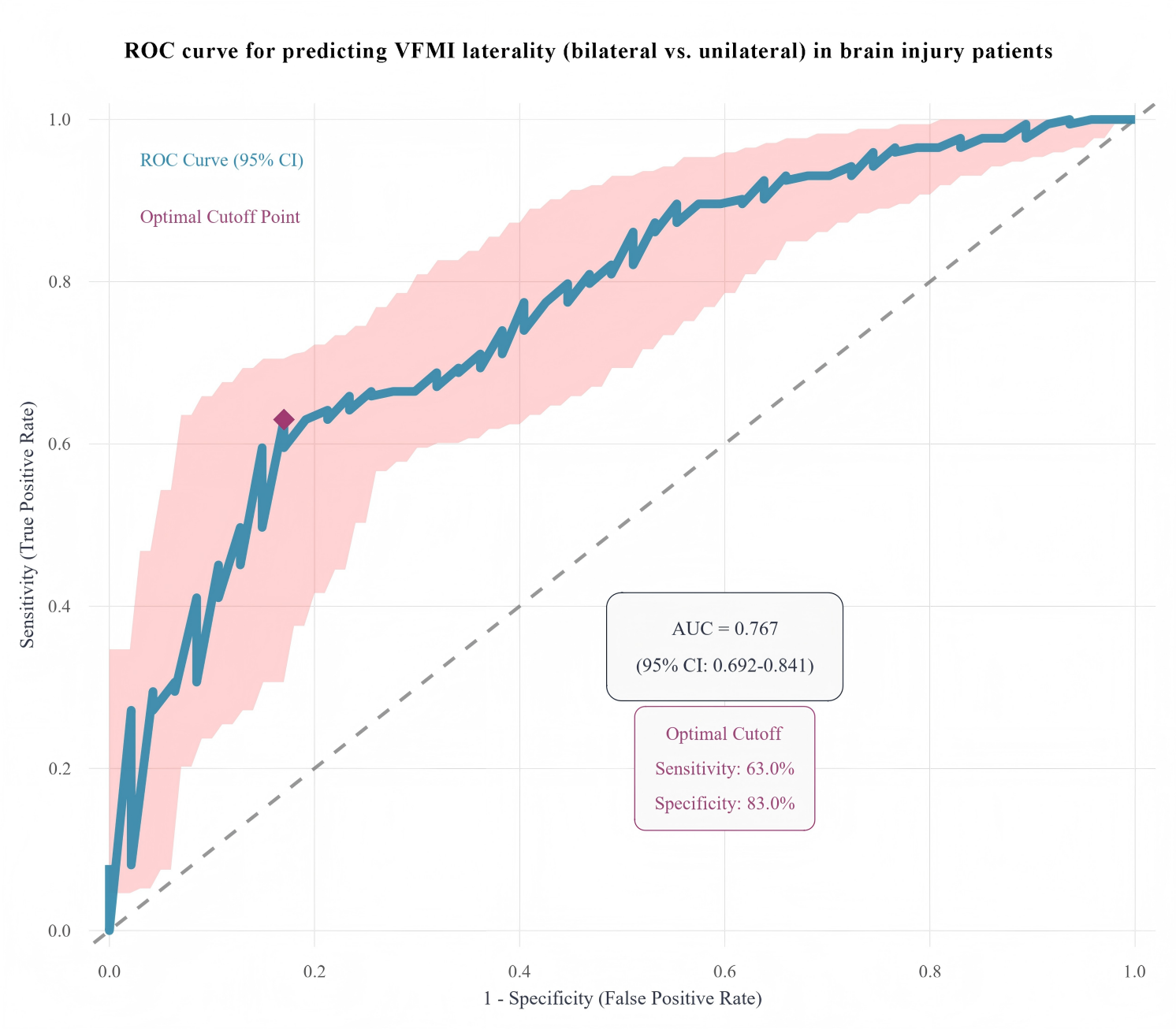
SupplementaryFigure 2.**AUC = 0.767 (95% CI: 0.692–0.841). Pink shading represents the 95% confidence band. The red diamond denotes the optimal cutoff point (sensitivity = 63.0%, specificity = 83.0%).

**Supplementary Figure3.**


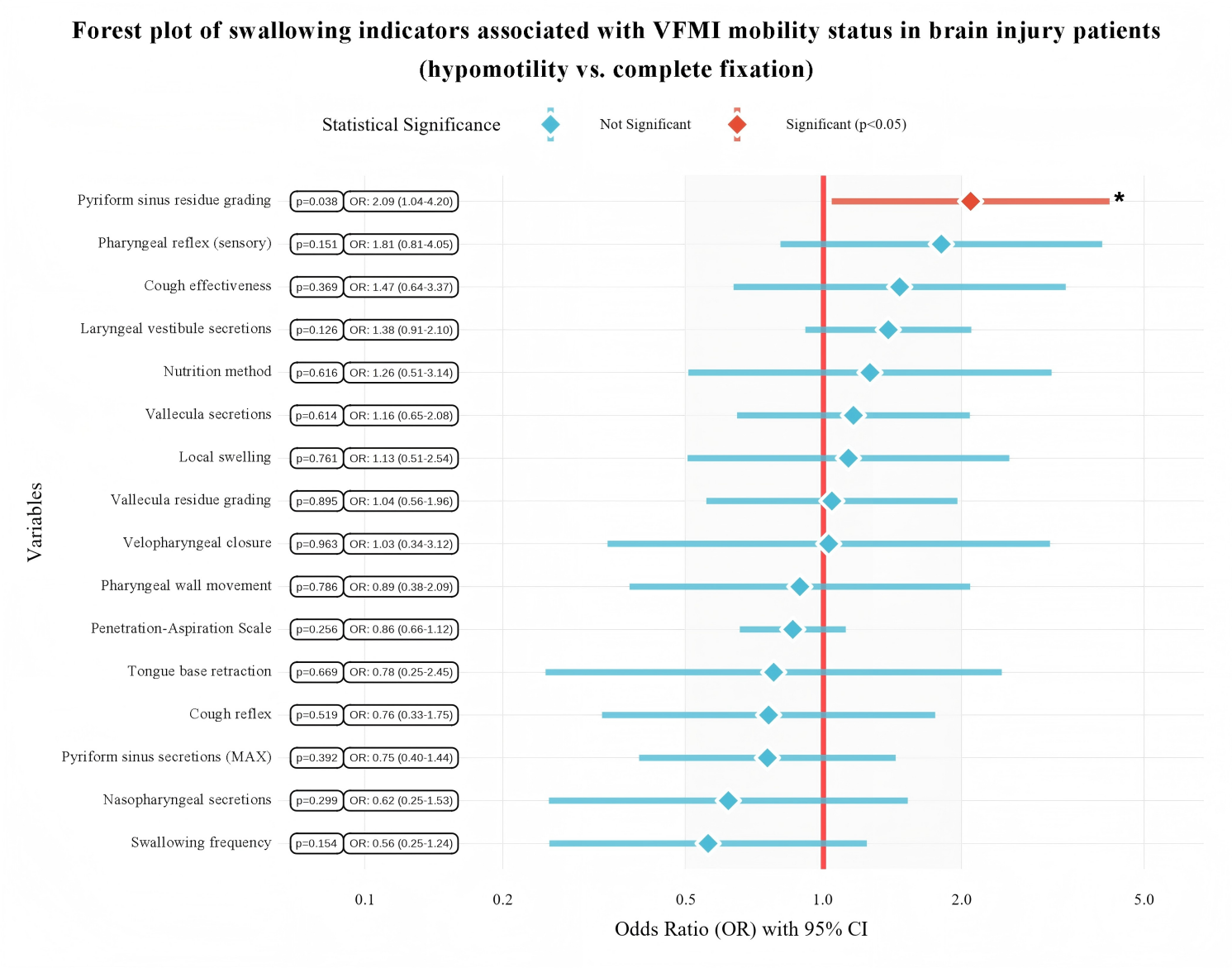


**Supplementary Figure 3.**Red diamonds indicate statistically significant variables (P < 0.05); blue diamonds indicate non-significant variables. Vertical red dashed line represents OR = 1.0.

**Supplementary Figure4.**


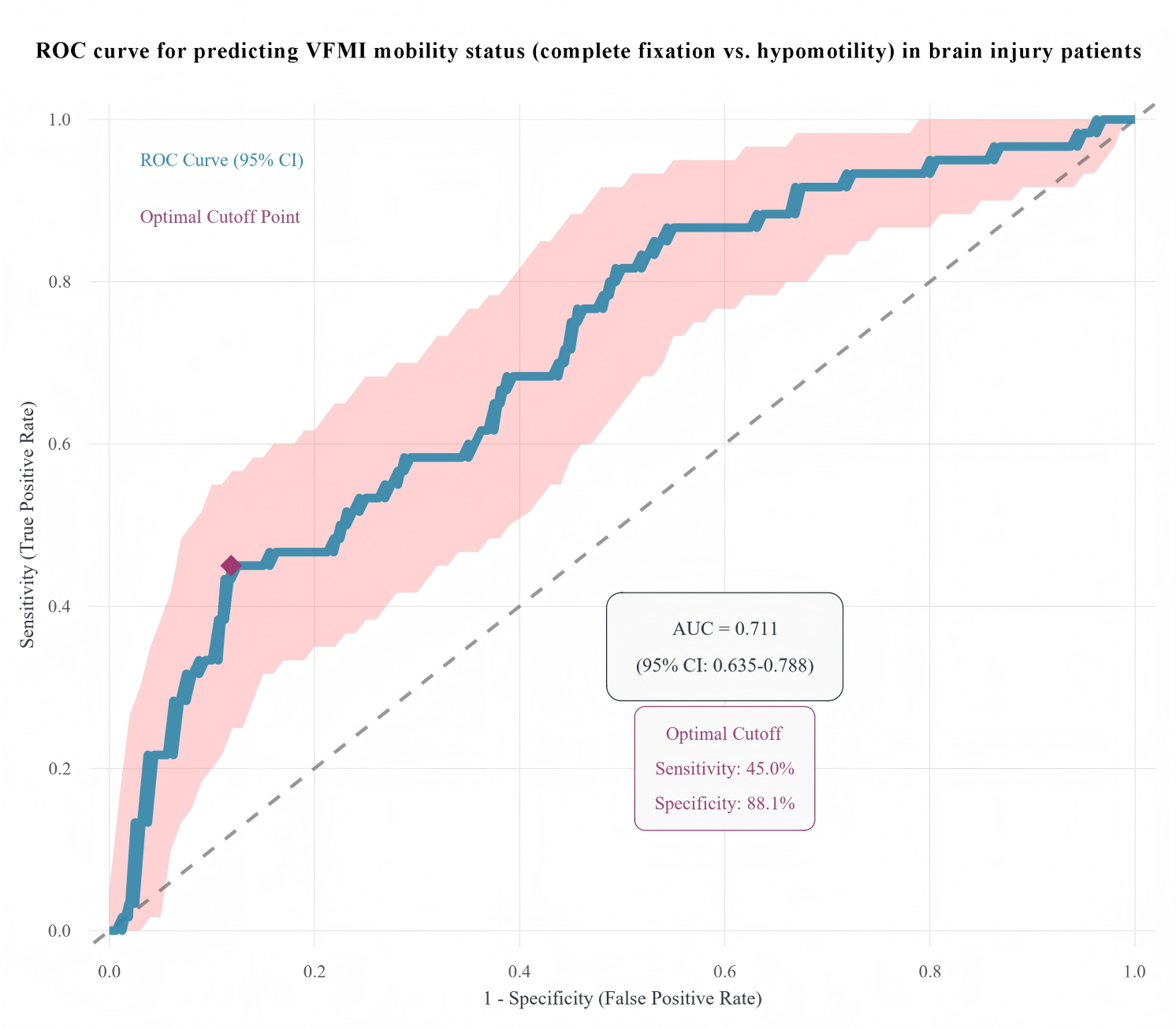


**Supplementary Figure 4.**AUC = 0.711 (95% CI: 0.635–0.788). The red diamond denotes the optimal cutoff point (sensitivity = 45.0%, specificity = 88.1%).
